# Comparison of THP‐1 Subtypes Reveals Differences on Transcriptomic and Protein Expression Level Throughout Their Immunological Differentiation

**DOI:** 10.1155/jimr/9427482

**Published:** 2026-08-17

**Authors:** Sabine Klatt, Gudrun Marquardt, Miriam Faxel, Stefan Rubner, Ioannis Papasotiriou

**Affiliations:** ^1^ Research and Development Department, Research Genetic Cancer Centre Central Europe GmbH, Weinbergweg 22, Halle (Saale) D-06120, Germany; ^2^ Research and Development Department, Research Genetic Cancer Centre International GmbH, Baarerstrasse 95, Zug CH-6300, Switzerland

## Abstract

Intensive usage of cancer cell lines as cellular model system in cancer research caused the generation of variant subtypes during the last decades. Genetic aberrations, altered transcriptomic profiles as well as changed protein expression have been reported for diverse cancer cell lines resulting in hampered reproducibility of experimental results within the scientific community. Remarkably, the human leukemic THP‐1 cell line, the most prominent model for immune cell research questions, was demonstrated to be a subject of branched evolution through diverse cultivation conditions; however, little is known about its impact on THP‐1 as monocyte model. This study compares the whole transcriptomic landscape of THP‐1 cells from two different depositors during differentiation into various immune states. Numerous genes showed significant altered transcript expression in immune‐related signaling pathways, which pervade through macrophage differentiation. These differences are reflected in key biological processes such as metabolic pathways, including glycolysis and lipid metabolism. Furthermore, analysis of immune cell surface markers by flow cytometry and determination of cytokine levels using LEGENDplex revealed variations in protein expression, such as increased interleukin 10 (IL‐10) raising questions about its impact on differentiation potential and immune function. These alterations have been evaluated by immune functional assays, including phagocytosis, determination of superoxide (SOX) levels as well as caspase‐1 activity. In addition, the transcriptomic comparison to a primary immune cell dataset revealed high similarity between the THP‐1 subtypes. However, minor discrepancies compared to primary cell types were identified, which could influence their immunological behavior. These findings suggest that the variability between THP‐1 subtypes may undermine the reliability of THP‐1‐based immunological research, especially when attempting to replicate experiments across laboratories.

## 1. Introduction

Reproducibility is a fundamental principle of scientific efforts and is aspired by the scientific community, enabling researchers to benefit from each other’s experience to forward the understanding of complex systems. However, reproducibility depends on data transparency, robust experimental design, and standardized laboratory practices. To obtain more comparable results, model systems are commonly used to address multifaceted and intricate biological problems. The use of cell models allows the investigation of biological processes at the cellular level within controlled laboratory environments. Increasing awareness of genetic instability in cell lines has raised concerns about the reliability and consistency of scientific findings based on these model systems. Notably, studies have shown that distinct laboratory practices can lead to the generation of subtypes within the same cell line, which may affect experimental results [1–5]. For instance, in 2004, Rintala‐Maki et al. [6] demonstrated that genomic evolution in the breast cancer cell model MCF7 can lead to different TNF‐*α* sensitivity through an unstable gene expression of LUCA‐15. Likewise, a large‐scale study analyzing multiple MCF7 subclones observed significant variability in drug responses and doubling times [7]. Similarly, long‐term cultivation of the human osteosarcoma cell line Saos‐2 resulted in changes in gene expression and morphology, including a reduction in decorin, which plays a key role in extracellular matrix organization and cell signaling [8]. These findings demonstrate how environmental and procedural factors, such as passage number and culture density, can significantly alter the cell behavior over time.

The human monocytic THP‐1 cell line, established in 1980 from a patient with acute monocytic leukemia (AML), is a widely used monocytic model for basic immunological research to study the role of monocytes during immune response [9]. Monocytes develop into macrophages, with different phenotypes such as proinflammatory M1 cells and M2 cells that promote tissue repair and immune suppression. They exist on a spectrum of activation states, balancing immune defense and tissue regeneration in response to varying stimuli. THP‐1 cells are widely employed in immunology because of the cells ability to differentiate into macrophages and dendritic cells [10–14]. Yet, despite their broad application, there is no consensus on standardized differentiation protocols for THP‐1 cells, often resulting in inconsistent outcomes. As with other cell lines, THP‐1 cells have undergone genomic changes over time, contributing to variability in their biological responses [15]. Kasai et al. [16] reported chromosomal divergence and transcriptomic variation among THP‐1 cells obtained from different depositors, which significantly affected their responses to phorbol‐12‐myristat‐13‐acetat (PMA)‐induced differentiation. Additionally, genetic drift in THP‐1 cells from different depositors has been linked to heterogeneity losses in key chromosomal regions, changes in human leukocyte antigen (HLA) type, and AML‐related gene expression profiles [17, 18]. Changes in culture conditions, including cell density prior to stimulation, can affect cell surface marker expression. For example, Aldo et al. [19] reported that THP‐1 cells cultured at higher densities showed increased CD14 expression following PMA treatment compared to cells seeded at lower densities.

Although numerous studies have documented variations in THP‐1 behavior due to genetic drift, depositor origin, or culture conditions, there is still very limited knowledge about how these variables influence the immune activation states of THP‐1‐derived macrophages. Given their status as a widely accepted immune cell model, a deeper understanding of the diversity within THP‐1 subtypes is essential. The present study aims to examine the differences in cell morphology, transcriptomic landscape, key metabolic processes, and protein expression levels, with particular emphasis on the various immune activation states of THP‐1 cells assessed by functional immune assays. Moreover, we compared our transcriptomic cellular setup to a primary immune cell data set. By highlighting the impact of subtype‐specific variability, we aim to contribute to a more reliable use of THP‐1 cells in immunological research and to raise awareness of how cell line evolution and subtype development can affect experimental reproducibility.

## 2. Materials and Methods

### 2.1. Cell Culture and THP‐1 Differentiation

THP‐1 cells were purchased from German Collection of Microorganism and Cell Cultures (DSMZ, Braunschweig, Germany) and European Collection of Authenticated Cell Cultures (ECACC, Salisbury, United Kingdom). Cells were maintained in RPMI 1640 medium (Thermo Fisher Scientific, 42401042) supplemented with 10 % heat‐inactivated fetal bovine serum (Thermo Fisher Scientific, A5256801), 1 % (v/v) penicillin/streptomycin (Thermo Fisher Scientific, 15140122), 1 % (v/v) L‐glutamine (Thermo Fisher Scientific, 25030024), 1 % (v/v) sodium pyruvate (Thermo Fisher Scientific, 11360039,) and 0.01 % (v/v) ß‐mercaptoethanol (Thermo Fisher Scientific, 31350010). Cells were cultured at 37 °C and 5 % CO_2_ in a humid environment. Cells were regularly tested negative for mycoplasma contamination during cultivation using TransDetect PCR Mycoplasma Detection Kit (Biotrend, FM311−01). Cells were verified by short tandem repeat (STR) analysis (Eurofins, Germany, Table S1). Cell number has been determined using automated brightfield cell counting (Countess, Thermo Fisher Scientific).

For THP‐1 differentiation, cells were seeded with a density of 120,000 cells/cm^2^ into different culture flasks containing media supplemented with 150 nM PMA (Sigma‐Aldrich, P8139) and incubated for 48 h. Cells were washed three times with RPMI media without FBS. Afterward, cells were rested for 24 h in culture media to generate M0 macrophages. For further polarization, cells were incubated for 72 h with RPMI containing 50 ng/mL each of interleukin 4 (IL‐4, Biolegend, 574006) and IL‐13 (Biolegend, 571106) for M2 macrophages or 100 pg/mL lipopolysaccharid (LPS, Sigma Aldrich, L6529) and 50 ng/mL interferon‐*γ* (IFN‐*γ*, Biolegend, 570204) for M1 macrophages.

### 2.2. Flow Cytometry Analysis

To detach adherent cells as M0 macrophages, M1 macrophages, and M2 macrophages, cells were washed twice with prewarmed PBS, followed by a 15 min incubation at 37 °C with Accutase (Capricorn, ACC‐1 B). The following steps were carried out at 4 °C with cold reagents. Cells were centrifuged and washed twice with PBS and resuspended in cell staining buffer (Biolegend, 420201). Nonspecific binding was blocked using human TruStain (Biolegend, 422302) for 15 min. Cells were stained with respective antibodies (including isotype controls) for 30 min (Table S2). Cells were washed three times in cell staining buffer before data acquisition within the CytoFLEX (Beckmann Coulter). FlowJo Software (commercial license) was used for data analysis.

### 2.3. LEGENDplex Analysis

To evaluate the cytokine levels, samples were collected at each differentiation state. Supernatant was centrifuged to exclude cellular debris and stored at ‐80 °C until further analysis. Supernatants were analyzed using LEGENDplex HU Essential Immune Response Panel (13‐plex) w/VbP Kit (Biolegend, 740930). Samples were prepared according to the manufacturer’s protocol. Data acquisition was performed using CytoFLEX (Beckmann Coulter). Data were analyzed using LEGENDplex software (BioLegend, San Diego, CA, USA).

### 2.4. Determination of Glycolytic and Lipid Metabolite Concentrations

For assessing metabolic activity, cell culture supernatants were collected at each differentiation state. The supernatant was then centrifuged to remove cellular debris and stored at −80°C until further analysis. The supernatants were analyzed using Glucose‐Glo (Promega, J6021), Lactate‐Glo (Promega, J5021), and Glutamine/Glutamate‐Glo (Promega, J8021) according to the manufacturer’s instructions.

To determine the intracellular cholesterol level, the Cholesterol/Cholesterol Ester‐Glo Assay (Promega, J3190) was used. Briefly, 20,000 cells per well were seeded in a 96‐well plate and differentiated. At the time point of analysis, the cells were washed twice with prewarmed PBS and then lysed with cholesterol lysis solution for 30 min at 37°C. The samples were then prepared according to the manufacturer’s protocol, and luminescence signal was measured using a microplate reader (Spark Multimode Microplate Reader, Tecan). To evaluate the fatty acid oxidation (FAO) potential of each differentiation state, the FAO‐Glo Fatty Acid Oxidation Assay (Promega, CS3271A08) was used. Briefly, media was removed, and cells were incubated with FAO substrate solution at 37°C for 60 min. Afterward, FAO detection reagent was added, and luminescence signal was determined within 10 min.

### 2.5. Determination of Caspase‐1 Activity, Phagocytosis Potential, and Level of Intracellular Superoxide (SOX)

For immune functional analysis, cells (20,000 cells per well, 96‐well format) were differentiated as described above.

For determination of general caspase‐1 activity in M0, M1, and M2 states, the Caspase‐Glo 1 Inflammasome Assay Kit (Promega, G9951) was used according to the manufacturer’s instructions. Briefly, after 5 min of equilibration to room temperature, cells were directly incubated with either Caspase‐Glo‐1 Reagent or Caspase‐Glo‐1‐YVAD‐CHO Reagent, which selectively inhibits caspase‐1, for 90 min in the dark at room temperature. Luminescence was measured using a microplate reader (Spark Multimode Microplate Reader, Tecan).

For evaluating the phagocytotic potential of THP‐1 derived macrophages, pHrodo Green *E. coli* BioParticles Conjugate for Phagocytosis (Thermo Fisher Scientific, P35366) were used. At M0, M1, and M2 states, cells were washed with prewarmed PBS and incubated with heat‐inactivated fluorescent labeled *E. coli* particles at 37°C. Fluorescence signal was measured every 15 min using a microplate reader at excitation/emission wavelengths of 509/533 nm for overall 90 min.

To assess intracellular levels of SOX after phagocytosis within M0, M1, and M2, the SOX detection kit (Abcam, ab139476) was used. Phagocytosis was induced as described above. The cells were then washed with washing buffer and incubated with the oxidative stress detection solution for 1 h at 37°C in the dark. The fluorescence signal was detected using a microplate reader at excitation/emission wavelengths of 550/610 nm.

### 2.6. Primary Immune Cell Isolation and Differentiation

For the isolation of primary human immune cells, fresh Leukopaks from healthy adult donors were purchased from Grifols Bio Supplies Inc. First, peripheral blood mononuclear cells (PBMCs) were isolated using the StraightFrom Leukopak PBMC Isolation Kit (Miltenyi, 130‐123‐456) in combination with the MultiMACS‐Cell24‐Separator‐Plus. Monocytes were isolated with a positive selection using CD14 MicroBeads (Miltenyi, 130‐050‐201) and differentiated using a standard protocol from Promocell. In brief, cells were seeded at 120,000 cells/cm^2^ in an appropriate cell culture flask with Monocyte Attachment media (Promocell, C‐28051). Cells were incubated at 37 °C and 5 % CO_2_ in a humid environment for 90 min. For further M0 macrophage differentiation, media was changed to macrophage generation media XF (Promocell, C‐28055, C‐28056) for 7 d, and for final polarization to either M1 or M2 macrophages, 100 pg/mL LPS (Sigma Aldrich, L6529) and 50 ng/mL IFN‐*γ* (Biolegend, 570204) or 50 ng/mL each IL‐4 (Biolegend, 574006) and IL‐13 (Biolegend, 571106) were added for 48 h, respectively. Cells were tested negative for mycoplasma contamination during cultivation using TransDetect PCR Mycoplasma Detection Kit (Biotrend, FM311‐01).

### 2.7. RNA Isolation for Gene Expression Analysis

RNA for transcriptome analysis during differentiation of THP‐1 cells was isolated via RNeasy Mini kit (Qiagen, 74106), including an additional on‐column DNase I digestion (Qiagen, 79256) according to the manufacturer’s instructions.

For primary monocytes and their corresponding macrophage states, cells were lysed using 1 mL QIAzol Reagent (Qiagen, 79306). 200 µL chloroform (Roth, 3313.1) per mL QIAzol was added, followed by homogenization of the suspension and centrifugation at 4°C, 12000x g for 15 min. The aqueous phase was merged with 1 volume cold isopropanol (Roth, CP41.1), and RNA was precipitated at – 20°C for 3 d. Precipitated RNA was pelleted at 4°C and washed three times with ice cold 75% ethanol (ITW Reagents, A3678). Afterward, the pellet was dried and dissolved in RNase‐free H_2_O.

### 2.8. Whole Transcriptome Analysis

Library preparation, sequencing, demultiplexing, and adapter trimming were performed by CeGaT GmbH (Tübingen, Germany). Sequencing libraries were generated using the Corall RiboCop (HMR/Globin) (Lexogen) protocol, following the manufacturer’s protocol. For all samples, 100 ng of total RNA was used as input. Libraries were sequenced on an Illumina NovaSeq X Plus system in paired‐end mode, yielding between 27 and 57 million paired‐end reads per sample with Q30 values ≥ 95.96 % across samples. Demultiplexing was performed with bcl2fastq v2.20, and adapter trimming was carried out using Skewer v0.2.2 [20].

Downstream bioinformatic analyses were conducted using an in‐house Nextflow v25.04.7 [21] pipeline. Reads were aligned to the human reference genome GRCh38 (Ensembl release 110) using HISAT2 v2.2.1 [22] with the parameter ‐‐rna‐strandness FR. Resulting BAM files were sorted and indexed with SAMtools v1.20. Deduplication was performed with UMI‐tools v1.1.6 [23]. Transcript‐level quantification was performed using HTSeq‐count v2.0.9 with the following parameters: ‐s yes ‐t exon ‐m intersection‐strict ‐‐nonunique = fraction.

All analyses were performed in R (v4.5.2). Raw transcript‐level counts were aggregated to gene‐level counts by summation. Genes with fewer than 1 count across all samples were removed prior to downstream analysis. For visualization, variance‐stabilizing transformations were applied using the regularized log (rlog) function from DESeq2 v1.48.1 [24]. For primary monocytes, two sample sets were generated. One for M0 before differentiation to M1 and one before differentiation to M2. Samples in both sets showed Pearson correlation coefficient ≈ 1.0 and were merged into a single group for all following differential expression analyses.

Differential expression analysis was carried out using DESeq2, including size‐factor normalization and dispersion estimation. Wald tests with apeglm shrinkage of log_2_ fold changes were applied, and significance was determined using Benjamini–Hochberg‐adjusted *p*‐values. Genes were considered significantly differentially expressed if they exhibited an absolute log_2_ fold change ≥ 1 or ≤ 1 and an adjusted *p*‐value ≤ 0.05. Uniquely expressed genes were identified by selecting genes with expression > 0.3 TPM in at least one sample of one condition, while all samples in the other condition have < 0.3 TPM.

The microarray analysis pipeline is described in detail in the Methodology section of the *Supporting Information*.

Enriched biological pathways were identified by using the gene set enrichment analysis package fgsea v1.34.2 [25] based on the hallmark gene sets by the Molecular Signature Database (MSigDb) [26, 27].

### 2.9. Statistical Analysis

All data are presented as mean ± standard deviation (SD). Flow cytometry data, LEGENDplex data, metabolite concentrations, caspase‐1 activity, and TPMs were analyzed using two‐way ANOVA with subsequent Tukey post hoc test for multiple comparison correction. Analyses were performed using GraphPad Prism 11 software (GraphPad Software Inc., La Jolla, CA, USA, commercial license). Statistically significant differences were set for *p*‐values ≤ 0.05 and are depicted in the respective diagrams.

## 3. Results and Discussion

### 3.1. Morphological Discrepancy of THP‐1 Subtypes During Differentiation to Immune States Is Accompanied by Changes on Transcriptomic Level

**Figure 1:**
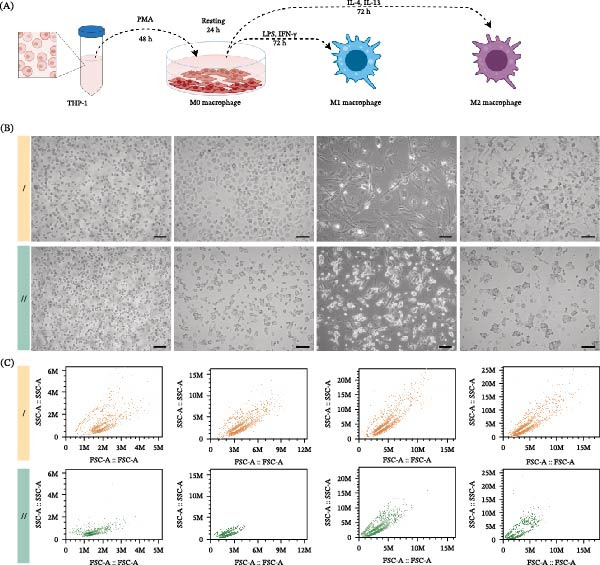
Subtype‐specific morphology of THP‐1 and its derived macrophages during differentiation process. (A) Schematic overview of the differentiation process of THP‐1 cells into distinct types of macrophages. (B) Representative brightfield images. Scale bar is set to 50 µm. (C) Flow cytometry analysis (forward vs. side scatter) of size and granularity of each cell type from depositor *I* and *II*.

In this study, THP‐1 cells obtained from two distinct depositors and designated as THP‐1_*I* and THP‐1_*II* were examined for their potential to differentiate into macrophages. We applied a defined protocol for directed and comprehensive THP‐1 differentiation which was adapted from Genin et al. [28]. Thus, the cells from both depositors were stimulated with PMA for differentiation into M0 macrophages and, subsequently, were further polarized either with LPS and IFN‐*γ* into M1 macrophages or with IL‐4 and IL‐13 into M2 macrophages (Figure 1A). The differentiation outcome can be monitored through their distinct morphological changes. Typically, THP‐1 cells are growing in suspension, while addition of PMA initiates cell adherence and, upon further polarization, cells either form spindle‐shaped protrusions or show a characteristic “fried‐egg” shape (Figure 1B).

Interestingly, under equal culture conditions a clear morphological distinction was already observed between THP‐1 cells obtained from two different depositors. THP‐1_*I* displayed a uniform, round morphology, whereas THP‐1_*II* appeared more oval‐shaped with small outgrowths, suggesting the evolution of distinct THP‐1 subtypes. Upon differentiation, these differences became more pronounced: M1_*I* and M2_*I* cells developed typical spindle‐like extensions and an adherent, flattened appearance, while M1_*II* and M2_*II* formed compact clusters of rounded cells (Figure 1B). Moreover, these visual differences were further supported by flow cytometric analysis of forward and side scatter characteristics, indicating variations in cell size and internal complexity (Figure 1C).

The phenomenon of cancer cell line evolution is mostly driven by genetic or epigenetic factors that can directly influence gene expression and cause alterations of cell function. These can lead to subclones with molecular features that confer fitness advantages, which is stochastically described by Hirsch et al. [29]. To explore these phenotypic differences at the transcriptomic level, we performed a whole transcriptome sequencing analysis, comparing both THP‐1 subtypes across their immune activation states (Figure 2). For validation, a microarray‐based approach was used in parallel to verify the data set, and similar results were obtained (Figure S1).

Figure 2Identification of different transcriptomic profiles of THP‐1 cells from both depositors. (A) Volcano plots visualize upregulated (red, p.adj. ≤ 0.05, log_2_(FC) ≥ 1) and downregulated (blue, p.adj. ≤ 0.05, log_2_(FC) ≤ –1) genes in each immune state comparing *II* to *I*. (B) VENN diagrams visualizing the overlap of upregulated and downregulated genes across different immune states between the two depositors. (C) Functional gene set enrichment analysis based on the Hallmark database revealing activated (red) and suppressed (blue) pathways for depositor *II* compared to *I*. (D) Heatmap presenting the log_2_(FC) of the inflammatory gene set for each differentiation state comparing depositor *II* to *I* with statistically significant difference displayed as star (p.adj ≤ .05).

**Figure:**
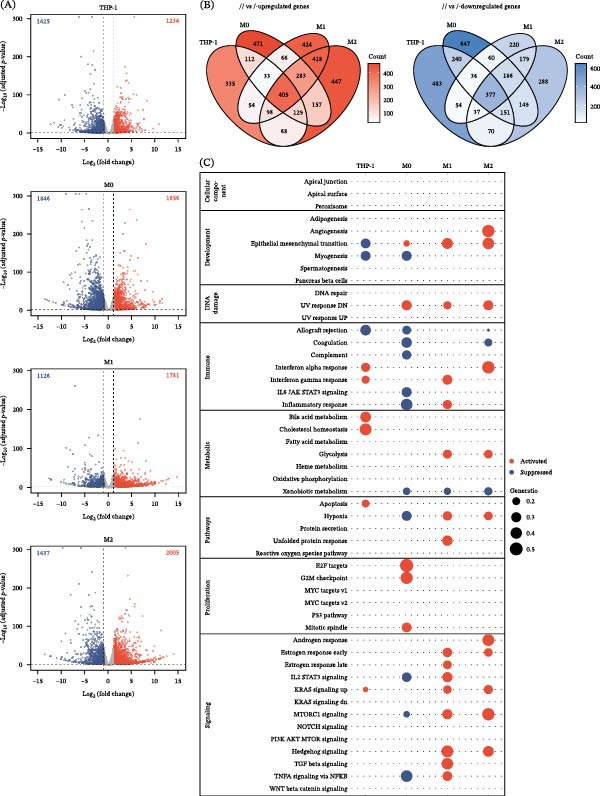

**Figure:**
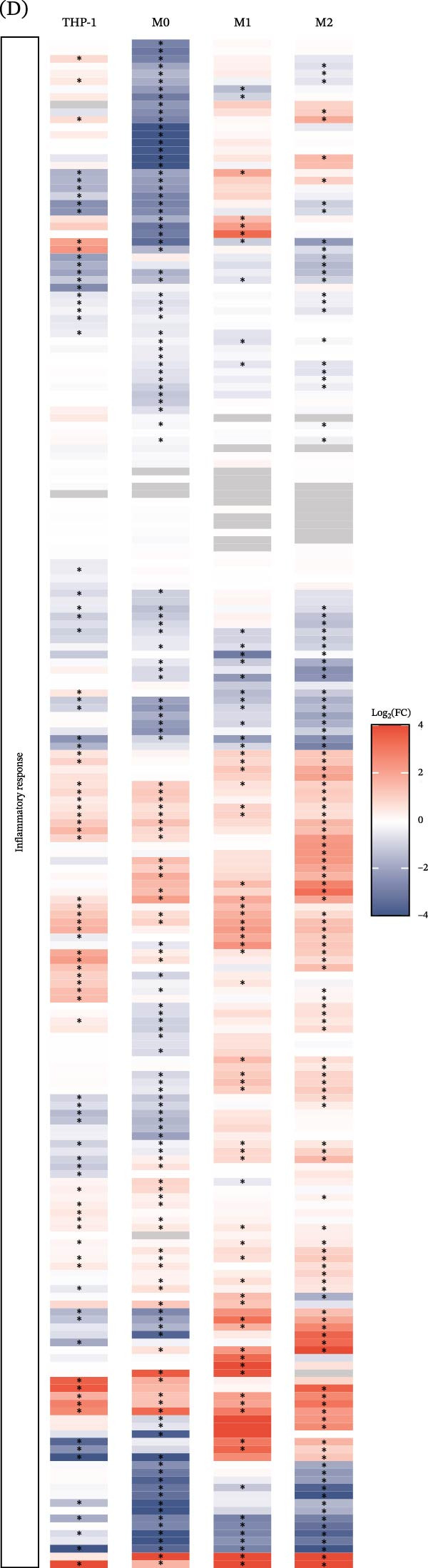

A total of 14,986 expressed genes, representing 89.8 % overlap, were detected in both subtypes, with the majority exhibiting similar expression levels. However, 1122 genes (7.5 %) or 412 genes (2.7 %) were exclusively transcribed in THP‐1_*I* or THP‐1_*II*, respectively (Figure S2). Moreover, differential gene expression was detected for 1425 downregulated and 1234 upregulated genes in THP‐1_*II* compared to THP‐1_*I*. These patterns persisted across all differentiation states, highlighting robust depositor‐specific gene expression profiles (Figure 2A), indicating that although both cell lines share a common origin, consistent transcriptional divergence has occurred.

The overlap of differentially expressed genes across immune states is summarized in Figure 2B, which reveals that only 12% of genes are consistently upregulated and downregulated across all states. Most transcriptional differences appear to be differentiation‐state‐specific. To better understand how these transcriptional differences affect cellular behavior, we conducted pathway enrichment analysis. Interestingly, no consistent trend was observed across pathways or pathway groups. Differential gene expression between THP‐1_*I* and THP‐1_*II* and their immune states was distributed across all eight major pathway categories, indicating a nondirectional, diversified shift in transcriptomic landscapes during differentiation. While relatively few pathway differences were observed between undifferentiated THP‐1 cells (9 pathways), substantial discrepancies emerged in M1 macrophages, where 16 pathways were either upregulated or downregulated depending on the depositor (Figure 2C). This suggests that divergence on RNA level between the subtypes becomes more pronounced during polarization toward proinflammatory states, possibly impacting their immunological functionality.

As the THP‐1 cell line is a common immune cell model, we closely inspected immune‐related gene sets. Analysis of the inflammatory response hallmark gene set (198 genes) showed significant variation between the two subtypes: 47 genes differed in undifferentiated cells, 82 in M0, 47 in M1, and 79 in M2 macrophages (Figure 2D). These findings suggest that not only the differentiation stage but also the cell source significantly influences the immunological potential of THP‐1 cells. Notably, we identified differential expression of two scavenger receptors of interest in immunology: macrophage receptor with collagenous structure (MARCO), which is downregulated in M0_*II* and M2_*II*, and macrophage scavenger receptor 1 (MSR1), which is predominantly expressed in those same differentiation stages. MARCO plays a crucial role in pathogen recognition and clearance by mediating opsonin‐independent phagocytosis, especially in response to LPS and lipoteichoic acid (LTA). Its expression is tightly regulated by cytokines such as IL‐1*α*, IL‐10, and IL‐6, with activation pathways involving p38 and STAT3 signaling [30]. MSR1, on the other hand, is critical for M2 macrophage polarization, and its activity can be reduced by cytokines such as TNF‐*α* and IFN‐*γ* [31]. Importantly, MSR1 participates in proinflammatory responses when activated by saturated fatty acids, triggering JNK signaling and promoting cytokine production (e.g., TNF‐*α*, IL‐6). The expression levels of MARCO and MSR1 in macrophages directly affect macrophage polarization, immune response regulation, and pathogen clearance. Thus, MARCO is more associated with immune surveillance and tolerance, while MSR1 influences proinflammatory responses and immune regulation, especially in the context of tumors [30]. These transcriptional differences may therefore have functional implications, potentially affecting how THP‐1‐derived macrophages respond to pathogens or inflammatory stimuli.

### 3.2. Glycolysis and Lipid Metabolism Differ in THP‐1 Subtypes During Differentiation

**Figure 3:**
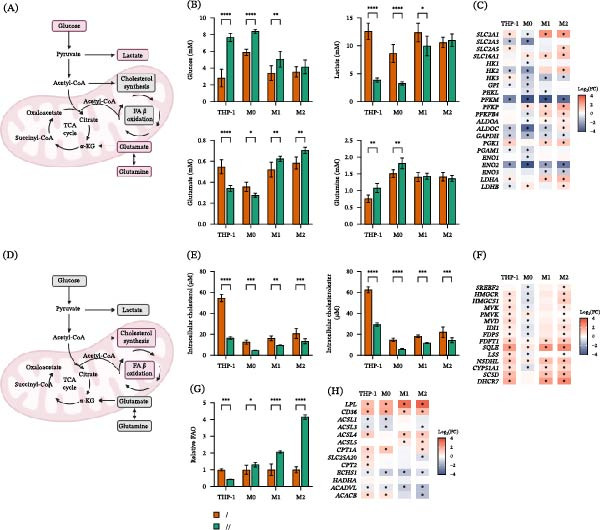
THP‐1 and its derived macrophages demonstrate significant differences in metabolic pathways regarding glycolysis and lipid metabolism. Scheme of key metabolites combining glycolysis and lipid metabolism highlighting either (A) glucose, lactate, glutamate, and glutamine or (D) cholesterol synthesis and fatty acid (FA) oxidation. Bar plots display glucose, lactate, glutamine, and glutamate concentrations in the medium (B), intracellular levels of cholesterol and cholesterol ester (E) and FAO rates relative to depositor *I* (G) for THP‐1 and its differentiation states. (C) Expression of key enzymes of glycolysis and lactate production, of cholesterol biosynthesis and its regulator SREBP2 (F) or of the fatty acid oxidation cascade (H) is presented as heatmaps. All heatmaps show the log_2_(FC) comparing depositor *II* to *I* for each differentiation state with statistically significant difference displayed as star (p.adj ≤ .05). Bar plots depict the mean ± SD (*n* = 4). Statistical significance was calculated using two‐way ANOVA with subsequent Tukey post hoc test for multiple comparison correction ( ^∗^ *p* ≤ .05,  ^∗∗^ *p* ≤ .01,  ^∗∗∗^ *p* ≤ .001,  ^∗∗∗∗^ *p* ≤ .0001). (A, D) Created in BioRender. Marquardt, G. (2026) https://BioRender.com/hhuy1x0.

Transcriptomic data revealed discrepancies in various biological processes, including metabolic pathways such as glycolysis and cholesterol metabolism, between the two subtypes. Consequently, we analyzed metabolic activity with relation to the differentiation states of the subtypes by determining the concentration of key metabolites, such as glucose, lactate, glutamine, and glutamate, within the supernatant of each immune state and transcript level of associated key enzymes (Figure 3A–C). Additionally, we investigated lipid metabolism by measuring intracellular cholesterol and cholesterol ester levels and the FAO potential, as well as corresponding gene expression patterns (Figure 3D–H).

THP‐1_*I*, M0_*I*, and M1_*I* exhibited higher glucose uptake from the medium, correlating with higher lactate export in the supernatant, which suggests enhanced glycolysis rates (Figure 3B). This difference equalized with differentiation to M2. Higher glucose uptake correlates with higher GLUT3 (SLC2A3) expression but not with GLUT1 (SLC2A1) expression (Figure 3C). GLUT1 showed an overexpression for M1_*II* and M2_*II* macrophages without a higher glucose uptake compared to M1_*I* and M2_*I*, suggesting the involvement of other post‐translational regulatory mechanisms. Several glycolysis enzymes, such as ENO2 and PFKM, showed elevated levels in subtype *I*, which supports the hypothesis of a higher glycolysis rate, especially in THP‐1_*I* and M0_*I* cells. With differentiation, it appears that cells from depositor *II* increased glycolysis and lactate production, reaching similar levels as depositor *I*‐derived cells. Interestingly, glucose appears to play an important role in inflammation processes. Freemerman et al. [32] demonstrated the direct effects of glucose transporter overexpression in a RAW264.7 cell model. The cells were in a hyperinflammatory state due to higher glucose uptake and metabolism, characterized by enhanced secretion of inflammatory mediators such as PAI‐1 and TNF‐*α*. Increased glucose utilization induces an ROS‐driven proinflammatory phenotype in macrophages, which may play an integral role in promoting obesity‐associated insulin resistance. Furthermore, inhibiting glycolysis in M1 macrophages impaired their function by interfering with phagocytosis, ROS production, and cytokine secretion [33].

A comparable trend was observed in glutamine and glutamate metabolite concentrations. THP‐1_*I* cells exhibited elevated glutamine consumption and glutamate production compared to cells from subtype II (Figure 3B). These discrepancies have been shown to become less pronounced during differentiation. Together, these observations underscore that subtype *I* exhibits elevated levels of general metabolic activity also reflected by transcript level of corresponding enzymes (Figure 3C).

Moreover, transcriptomic analysis revealed significant subtype‐specific differences in cholesterol metabolism. Closer examination of intracellular cholesterol and cholesterol ester levels indicated higher levels of intracellular cholesterol in subtype *I*, and a similar trend was observed during differentiation, with the gap decreasing as differentiation progresses (Figure 3E). THP‐1_II exhibited only 30 % of the cholesterol level detected in THP‐1_*I*, aligning at around 60 % in M1 and M2 cells. As illustrated in Figure 3E, intracellular cholesterol ester levels increased in subtype *I*,equally. Differences in cholesterol levels are important for immune function, especially in the context of atherosclerosis and tumor‐associated macrophages [34, 35]. The overall higher cholesterol in cells from depositor *I* leads to an expected reduced expression of HMGCR, HMGCS1, MVK, and SQLE, which as targets genes of sterol regulatory element‐binding transcription factor 2 (SREBP2) are involved in cholesterol biosynthesis. Only M0 macrophages, displaying a higher expression for cholesterol biosynthesis enzymes and its regulator SREBP2 (Figure 3F), also encompassed a higher cholesterol content (Figure 3E).

Furthermore, an examination of the FAO rates revealed that THP‐1_I cells exhibited significantly higher activity. This process was characterized by a shift in differentiation that led to higher levels of FAO in the M0_II, M1_II, and M2_II differentiation states. Notably, M2_II cells exhibited a fourfold higher FAO activity (Figure 3G). A major characteristic of M2 macrophages is the use of FAO to break down fatty acids in order to produce energy, which indicates a more typical M2 phenotype for depositor II. The differences in FAO rates only slightly correlated with the differentially expressed enzymes involved in lipid uptake (LPL and CD36), processing (ACSL family, CPT1A, SLC25A20, and CPT2), and *β*‐oxidation (ECHS1, HADHA, and ACADVL) (Figure 3H). The discrepancy may be due to the higher ACC2 (ACACB) expression in THP‐1_II and M0_II. ACC2’s product, malonyl‐CoA, inhibits CPT1A function [36]. Further regulation could be mediated by intracellular acetyl‐CoA pools and the possible acetylation of enzymes and histones, which serve as additional translational regulation.

Overall, our results show a subtype‐specific behavior of energy processing especially in undifferentiated THP‐1 and early differentiated M0 macrophages. While polarization of macrophages in M1 and M2 states promote an alignment of glycolysis and cholesterol metabolism between the two subtypes, differences in FAO arise.

These variations must be considered, especially when assessing the impact of the tumor microenvironment. This is particularly noteworthy given that THP‐1‐derived cells are often used to mimic tumor‐associated immune cells in coculture models. The metabolic activity of different cell types contributes to shaping the tumor microenvironment. Consequently, it is crucial to consider the variations that may emerge when employing distinct THP‐1 subtypes [37]. Furthermore, drugs that target metabolic pathways to inhibit specific subsets have been explored as novel therapeutics for macrophage‐related diseases [38], which highlights the importance of metabolic characterization.

### 3.3. Subtype‐Dependent Expression of Immune‐Related Cell Surface Markers and Cytokine Levels

The combination of depositor‐specific characteristics in terms of morphology, transcriptome, and differences in metabolic activity raises the question whether such divergence also manifests at protein expression levels for immune‐relevant surface markers. To address this, we employed flow cytometry to analyze key surface antigens across all differentiation states in both THP‐1 subtypes (Figure 4). Specifically, we focused on key immune‐related markers including CD86, CD206, CD14, and CD209, known to indicate macrophage identity and activation state. Evaluating the protein expression of these markers via flow cytometry provides valuable insight into the functional immunological properties of the macrophage populations derived from each depositor and whether transcriptomic divergence translates into phenotypic and functional differences.

Figure 4Different protein abundance of immune‐related cell surface markers during differentiation compared to mRNA levels. (A–D) mRNA expression levels based on TPMs. (E–H) Representative flow cytometric histograms visualize the percentage of cells presenting the cell surface markers CD86, CD206, CD14, and CD209. The corresponding isotype controls are shown as dotted lines. Bar charts depict the mean ± SD (*n* = 4). Statistical significance was calculated using two‐way ANOVA with subsequent Tukey post hoc test for multiple comparison correction ( ^∗^ *p* ≤ .05,  ^∗∗^ *p* ≤ .01,  ^∗∗∗^ *p* ≤ .001, ^∗∗∗∗^ *p* ≤ .0001).

**Figure:**
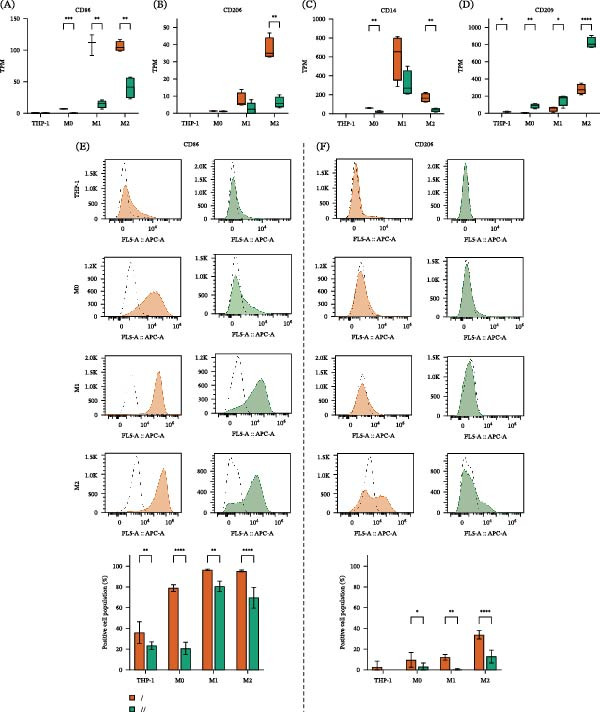

**Figure:**
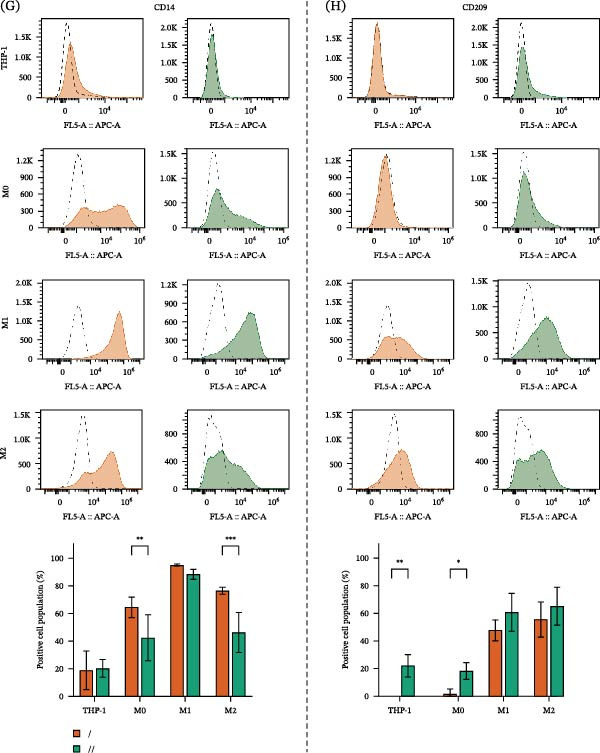

When examining the immune markers CD86, CD206, CD14, and CD209, the data revealed notable differences in cells from the two depositors. While the expression of general immune cell markers like CD16, CD45, CD32, and CD163 showed similar protein expression patterns in cells from different depositors (Figures S3 and S4A,C). CD86 protein expression was significantly elevated in M0_*I*, while M0_*II* displayed only a modest increase (Figure 4E). This difference is also reflected at gene expression level, where M0_*I*, M1_*I*, and M2_*I* showed elevated TPM values, but interestingly, while the biggest differences on RNA level were observed in M2 cells, on the protein level the biggest difference appeared in M0 cells (Figure 4A). These variations are critical because expression levels of CD86 can significantly influence immune responses. High CD86 expression is typically associated with M1 macrophages, while low expression is linked to M2 macrophages [39]. CD206 (mannose receptor) is a marker for M2 macrophages, and its expression, along with CD86, is used to distinguish between M1 and M2 macrophage phenotypes in tumors [40]. CD206 showed a robust increase in M2_*I*, consistent with both RNA and protein data (Figure 4B,F). CD14 protein expression also varied significantly between subtypes. THP‐1_*I* displayed elevated CD14 expression in all immune states (M0, M1, and M2), while M0_*II* and M2_*II* showed only moderate induction (Figure 4C,G). CD14 is involved in LPS signaling, influencing nucleic acid uptake in macrophages and modulating cytokine production, such as IL‐6 [41]. CD209 (DC‐SIGN) showed similar protein‐level shifts, with slightly elevated expression in subtype *II*, which is further reflected on transcriptomic level (Figure 4D,H).

Together, these findings emphasize the functional consequences of differences in surface marker expression among subtypes. Even when identical differentiation protocols were applied, THP‐1 subtypes from different depositors result in macrophages with different immunophenotypes. This variability must be considered when interpreting data from immunological studies, especially those related to macrophage plasticity, polarization, and immune modulation.

**Figure 5:**
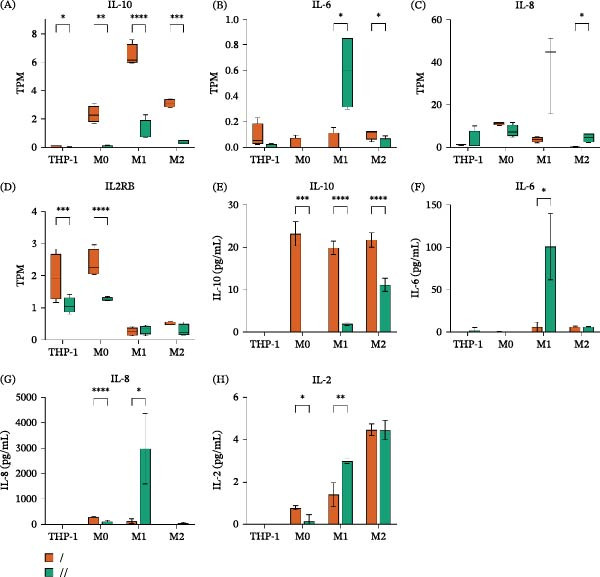
Quantification profiles of secreted analytes in the supernatant during differentiation compared to mRNA levels. (A–D) mRNA expression level based on TPMs. (E–H) Concentrations of secreted analytes in supernatant samples. Bar charts depict mean ± SD (*n* = 4). Statistical significance was calculated using two‐way ANOVA with subsequent Tukey post hoc test for multiple comparison correction ( ^∗^ *p* ≤ .05,  ^∗∗^ *p* ≤ .01,  ^∗∗∗^ *p* ≤ .001,  ^∗∗∗∗^ *p* ≤ .0001).

Moreover, it is important to assess whether similar variations extend to other immune‐related components, such as cytokines, which are key players in inflammation and serve as important signaling molecules in the immune system. To investigate this further, a human essential immune response panel was applied to analyze immune‐related analytes using flow cytometry in the corresponding supernatant (Figure 5).

In general, a correlation between mRNA levels and corresponding protein concentrations is observed across most analytes, accompanied by prominent depositor‐dependent phenotypic variations. After differentiation is induced by PMA, a robust secretion of IL‐10 is detectable in M0_*I*, M1_*I*, and M2_*I* cells (Figure 5A,E). In contrast, IL‐10 levels in THP‐1_*II* cells remain at or below the detection limit across all immune states except M2. This divergence highlighted the intrinsic differences between the two subtypes, affecting cytokine gene regulation. The elevated IL‐10 secretion during the differentiation of THP‐1_*I* suggests a more pronounced immunoregulatory phenotype, potentially making these cells more pronounced anti‐inflammatory macrophage subtypes in certain disease contexts. IL‐6 also exhibited a depositor‐dependent pattern (Figure 5B,F). In M1_*II*, IL‐6 protein levels were higher, which correlated with elevated CD14 expression (Figure 4C,G). This result aligns with other literature where the blocking of CD14 in monocytes and macrophages was linked to a 50% reduction of IL‐6 secretion [42]. Due to IL‐6’s pivotal role in inflammation and immune activation, this discrepancy has implications for the reproducibility of experiments and highlights the influence of subtype variation on the interpretation of inflammatory responses. Detection capability of IL‐6 could be influenced by different signaling pathways of IL‐6, influencing the availability of free, measurable IL‐6. High‐affinity cytokine binding exhibits fast association and slow dissociation kinetics, reaching equilibrium within seconds to minutes, which has a crucial impact on IL‐6 detection [43].

As seen for IL‐6, an elevated level of IL‐8 was only observed in M1_*I*, which correlates with the highest gene expression observed in M1_*I* (Figure 5C,G). In contrast, its receptor CD182 (Figure S4B,D) showed the highest expression in undifferentiated THP‐1 cells. An explanation for this could be the differential regulation of CD182, likely due to IL‐8‐driven receptor downregulation mechanisms. Given IL‐8’s dual role in promoting inflammation and modulating immune cell trafficking, such divergent expression patterns could profoundly impact studies of chemotaxis and immune cell activation. For example, prolonged IL‐8 secretion may reduce CD182 expression, thereby decreasing functional responses, such as monocyte tumoricidal activity, as reported in previous studies [44].

Notably, IL‐2 expression and its associated receptor, IL2R, further demonstrated the nuanced interplay between free cytokine levels and receptor binding. While both subtypes show high gene expression of IL2R in undifferentiated THP‐1 cells, no free IL‐2 has been detected within the supernatant (Figure 5D,H). By induction of differentiation processes, the receptor expression is reduced aligning with elevated IL‐2 levels in M0, M1, and M2 cells. This has further been described by Deng and Mosmann [45] analyzing individual cytokine dynamics in multiplex assays.

While the analytes in Figure 5 capture key aspects of cytokine regulation, Figure S5 provides broader insight into other immune markers, including TGF‐*β*1 and IL‐17 A. For TGF‐*β*1, higher concentrations were observed in the supernatant of M0_*I*, which may suggest that these cells exhibit a more rapid response to differentiation stimuli compared to THP‐1_*II*.

The patterns described here emphasize the multifaceted complexity of cytokine regulation and the critical influence of cell line origin. IL‐6 and IL‐10 serve as prominent examples of how inflammatory and anti‐inflammatory responses are modulated not only by the differentiation state but also by characteristics of the THP‐1 subtype. IL2R and CD182, as receptors mediating interleukin‐driven responses, further highlight the interconnected regulation between cytokines and their signaling partners. Dysregulation of these pathways may have physiological consequences, especially in models of infection, cancer, and autoimmunity.

Importantly, these observations reinforce the broader concept that transcriptomic data, while invaluable for identifying regulatory trends and signaling pathways, do not consistently correlate with protein level. Factors such as mRNA stability, translation efficiency, and protein turnover can all influence protein abundance [46]. For instance, mRNAs can be degraded rapidly, sequestered into translationally inactive forms, or translated at varying rates depending on cellular conditions. Furthermore, protein abundance is also modulated by degradation pathways like the ubiquitin‐proteasome system or feedback loops that regulate synthesis [47]. In general, differences between cytokine levels and transcriptomic data are not unusual. While transcriptomic data reflects mRNA at a single time point, supernatant‐based assays measure cumulative cytokines over time. Additionally, cytokine dynamics may vary due to receptor binding and internalization rates, which could lead to over‐ or underestimation of actual cytokine levels [48]. Nevertheless, transcriptomic data remains a powerful tool for detecting regulatory trends, identifying pathway activation, and highlighting potential targets. When interpreted alongside proteomic and functional assays, they contribute significantly to understanding cellular behavior and immune potential.

Finally, these findings support the notion that long‐term culturing and handling differences at depositor facilities contribute to subtype divergence in continuous cell lines. These differences are not technical artifacts but have biological significance, as seen in the divergent cytokine profiles of the THP‐1 subtypes. The marked expression differences suggest subtype‐specific responsiveness to pathogen‐associated stimuli and may affect downstream signaling cascades. This underlines the necessity of standardizing cell sourcing and validating cell lines functionally.

### 3.4. Immune Functional Characterization of THP‐1 Subtype‐Derived Macrophages

**Figure 6:**
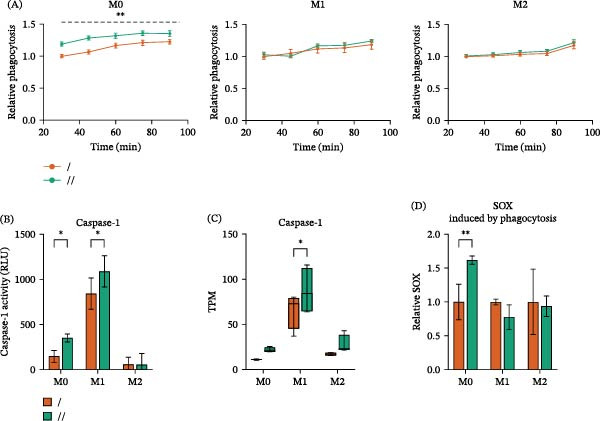
Assessment of immune function regarding phagocytosis, caspase‐1 activation and SOX production. (A) Phagocytosis activity over a time course of 30 to 90 min after phagocytosis induction relative to depositor *I* for all three differentiation states. Statistical significance was calculated using two‐way ANOVA with statistical significance depicted as stars ( ^∗^ *p* ≤ .05,  ^∗∗^ *p* ≤ .01,  ^∗∗∗^ *p* ≤ .001,  ^∗∗∗∗^ *p* ≤ .0001). (B) Caspase‐1 activity in all three differentiation states. (C) Caspase‐1 mRNA expression depicted as TPMs. (D) By phagocytosis induced SOX production relative to depositor *I*. Bar charts depict mean ± SD (*n* = 4). Statistical significance was calculated using two‐way ANOVA with subsequent Tukey post hoc test for multiple comparison correction ( ^∗^ *p* ≤ .05,  ^∗∗^ *p* ≤ .01,  ^∗∗∗^ *p* ≤ .001,  ^∗∗∗∗^ *p* ≤ .0001).

Next, we investigated whether subtype variations impact the immune functionality of the cell model. Thus, phagocytosis, caspase‐1 activity, and SOX levels were determined for M0, M1, and M2 macrophages (Figure 6).

A comparison of the capacity for cellular uptake of debris and pathogens of macrophages in M0, M1, and M2 states between the two subtypes revealed a discrepancy in the M0 state (Figure 6A). M0_*II* exhibited a higher fluorescence signal than M0_*I*, which was associated with a greater number of particles that had been absorbed over time. This trend was observed across all time points in the time series. However, no significant variations were observed in the M1 and M2 immune states, indicating a similar phagocytotic potential.

Furthermore, caspase‐1 exhibited markedly elevated activity in subtype *II* differentiation states. As part of the NLPR3 inflammasome, caspase‐1 acts as a signaling platform that activates inflammatory caspases, triggering innate immune responses. Generally, a similar trend is observed between the two subtypes, but there are differences in how strongly they appear (Figure 6B). Comparing the caspase‐1 activity to the transcriptomic level of the CASP‐1 gene revealed a strong correlation between higher TPM values for subtype *II* in all states and elevated caspase‐1 activity in M0_*II* and M1_*II* (Figure 6C). Qian et al. [49] employed a conditioned medium (CM)‐based approach combined with transcriptomic profiling and in vivo xenograft experiments to model tumor cell‐macrophage communication. Based on CASP1 knockdown experiments in THP‐1 and MOLM‐13 cells resulting in secretome shifts, they hypothesized that CASP1 contributes to an immunosuppressive microenvironment in AML. Immunohistochemical analysis revealed a remodeled microenvironment with reduced proliferation, increased EGR4, and suppressed IL‐10/p‐STAT3 and CD206 pathways, alongside increased CD86.

In accordance with phagocytosis, analyzing the level of produced SOX revealed a higher level in M0_*II* than in subtype *I* (Figure 6D). The potential to produce reactive oxygen species such as SOX is a key parameter that is frequently employed in immune‐related cell models. One notable application is the usage in cocultivation studies with cancer cells to investigate the protumorigenic impact of macrophages. Kovaleva et al. [50] demonstrated that PC‐3/DU‐145 cell lines became more resistant to macrophage cytotoxicity, exhibited faster doubling times, and displayed enhanced tumorigenic potential through repeated cocultivation with immune cells.

Together, these findings emphasize significant differences in immune functionality in the M0 state. Meanwhile, M1 and M2 macrophages exhibit similar phagocytic potential and levels of SOX, only showing significant differences for caspase‐1 activity in M1 state. Different macrophage states are relevant to different immunological questions, and this should be considered. In general, M1 macrophages are used in studies acting as proinflammatory cells, while M2 macrophages are considered in experimental setups investigating wound healing and tissue repair. Unpolarized macrophages, such as M0, are commonly exploited as an initial state in immunological research and analyze the differentiation potential of toxic substances or natural products. For example, Shah et al. [51] investigated the differentiation shift in THP‐1‐derived macrophages exposed to flavored e‐liquids.

These data support our hypothesis that THP‐1 and immune cell model characterization are necessary for each research question and should be considered separately. For example, they are important for studying organoids in the context of cell interactions and therapeutic responses, such as chemotherapy [52].

### 3.5. Transcriptomic Comparison of THP‐1 Subtypes as Immune Cell Model to Its Primary Counterpart

Considering all observed differences on cell morphology, transcriptome, protein expression, metabolic state, and immune‐related responses between the two THP‐1 subtypes, an important question arises: Does one of the subtypes more closely resemble primary human immune cells? Addressing this question is essential as THP‐1 cells are widely used as a representative model for monocyte‐derived macrophages in immunological research. In order to obtain initial indications, monocytes were isolated from fresh Leukopaks of healthy donors and differentiated into macrophages under standardized conditions. Transcriptomes at each differentiation state were bioinformatically examined upon RNA sequencing (Figure 7), and results were validated via microarray analysis (Figure S6).

Figure 7Transcriptome comparison of THP‐1 immune cell model to its primary counterpart identifying a depositor independent overlap. (A) Pearson correlogram of all samples indicating high correlation in dark blue and hierarchical clustering of the samples. (B) VENN diagrams showing overlap of expressed genes in primary immune cells (gray), depositor *I* (orange) and depositor *II* (green). (C) Gene set enrichment analysis based on hallmark gene sets showing activated (red) and suppressed (blue) pathways in the comparison of depositor *I* or *II* to primary immune cells. (D) Heatmap of inflammatory response pathway gene set in depositor *I* and *II* compared to the primary set‐up with statistically significant difference displayed as star (p.adj ≤ .05).

**Figure:**
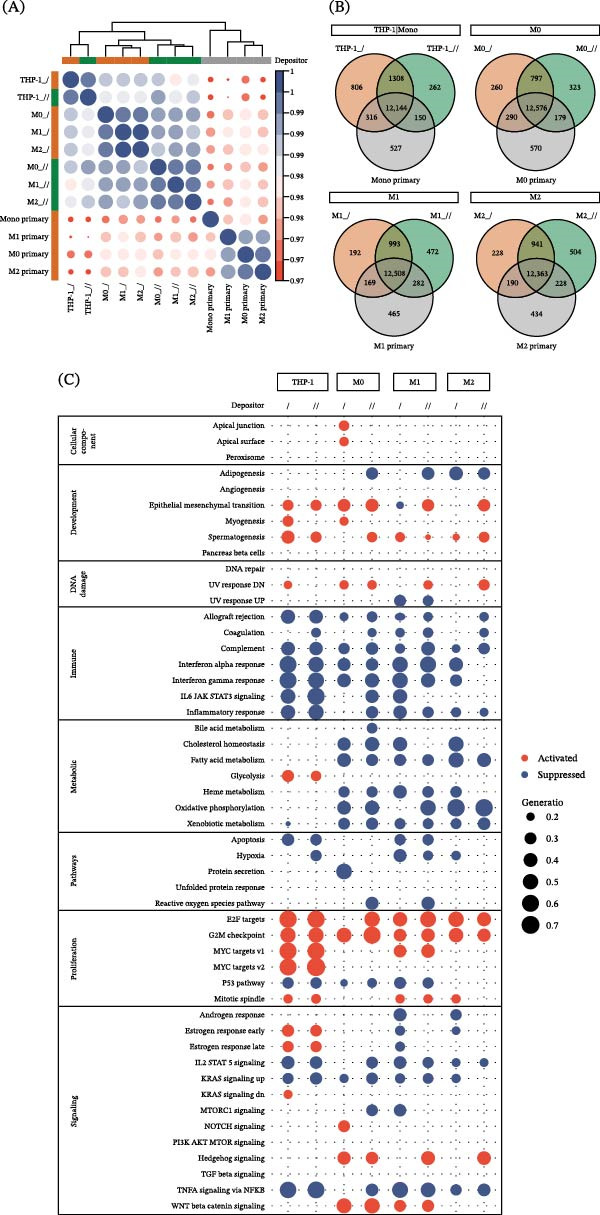

**Figure:**
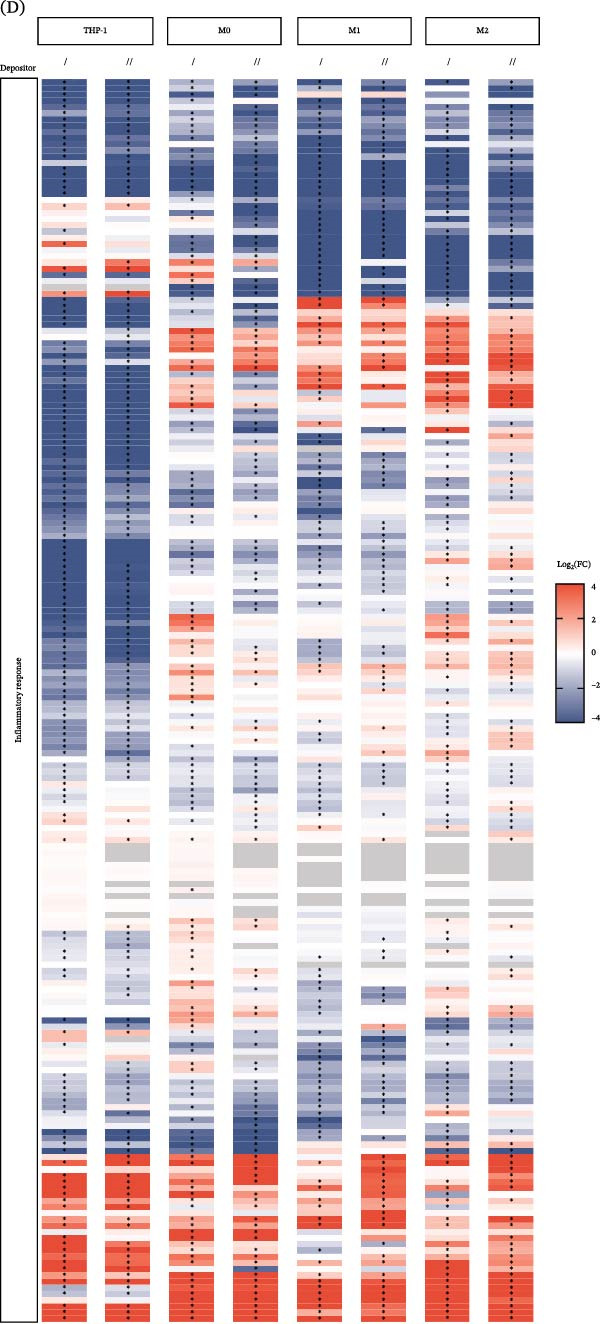

This primary immune cell data set was then compared to both THP‐1 subtypes across the distinct immune states (undifferentiated, M0, M1, and M2), allowing a comprehensive evaluation of how well the gene expression profile of each THP‐1 variant reflects its human counterpart (Figure 7).

Our analysis revealed that, compared to the differences between THP‐1 and primary cells, the variations between depositor subtypes of THP‐1 were minor. This was particularly evident in the correlogram, which showed strong transcriptomic correlations between THP‐1_*I* and THP‐1_*II* cells across all examined differentiation states (Figure 7A). The most prominent transcriptomic divergence occurred between THP‐1 cells and primary monocytes/macrophages, regardless of depositor origin. However, the differentiation process led to a closer approximation of THP‐1 gene expression profiles to those of primary immune cells, with differentiated THP‐1 states aligning more closely with their respective differentiated primary counterparts. Similarly, a PCA plot shows a clustering of the THP‐1 cells and a clear difference to primary monocytes (Figure S7). For the differentiation states, a similar shift of the transcriptome can be observed but no clustering to the primary macrophages. This supports the concept that differentiation enhances the biological relevance of THP‐1 cells as a model system. Underlining this observation, Venn diagram analyses revealed a high overlap in expressed genes between both THP‐1 subtypes and the primary cells, ranging from 78 % to 84 % depending on the immune state (Figure 7B).

To gain further insight into functional differences, we performed gene set enrichment analysis using hallmark gene sets. Both THP‐1 subtypes showed largely comparable patterns of upregulated and downregulated pathways when compared to primary immune cells (Figure 7C). Nonetheless, some pathway‐specific deviations became apparent. For example, myogenesis was upregulated in THP‐1_*I* and M0_*I* but not in THP‐1_*II and* M0_*II* cells. A similar trend was observed for the epithelial mesenchymal transition (EMT). While the pathway was upregulated in both THP‐1_*I* and THP‐1_*II*, as well as M0_*I* and M0_*II*, compared to the primary cells, further upregulation was evident only in M1_*II* and M2_*II*. These findings reflect narrow yet biologically relevant differences in how each THP‐1 subtype responds to differentiation stimuli and suggest that some pathways may be more sensitive to underlying genomic or epigenetic variability. While these variations may not undermine the general utility of THP‐1 cells as a model system, they highlight the importance of carefully considering cell line origin in pathway‐specific investigations.

Taking a closer look at immune‐related pathways, the inflammatory response gene set revealed similar trends between THP‐1 cells from both depositors and primary immune cells. Heatmaps of differential gene expression comparisons between THP‐1 and primary cells for the inflammatory pathway indicated largely congruent patterns, with only minor discrepancies between depositors (Figure 7D). These results indicate that both THP‐1 subtypes maintain important immunological functions, especially after differentiation.

Our transcriptomic comparison confirmed that the THP‐1 cell line exhibits a high degree of similarity to primary immune cells, particularly after differentiation, regardless of the depositor. This suggests that both depositors provide reliable models for studying macrophage and monocyte biology. However, minor discrepancies, such as differences in cell surface marker expression and cytokine profiles, highlight the importance of considering these factors when selecting THP‐1 cell lines for immunological applications. Owing to their ability to mimic certain aspects of human immune responses, immortalized cell lines like THP‐1 cells remain invaluable tools in both basic and advanced immunological research. They offer practical advantages, such as ease of use, cost effectiveness, and the absence of ethical concerns associated with animal research. However, it is important to recognize that these models are simplified representations of human physiology. Primary cells, while more difficult to maintain and subject to high donor‐dependent variability and ethical constraints, provide a closer approximation of in vivo conditions. They are essential for understanding immune mechanisms and testing therapies. Advanced models, such as 3D cultures and fluidic systems, assist in addressing key limitations, including immune system complexity and cancer heterogeneity [52–55]. Researchers must be cautious about the differences between cell models and actual human immune responses when applying data to broader biological contexts.

## 4. Conclusion

This study investigated discrepancies observed in THP‐1 cells obtained from two distinct cell banks. The analysis included a comprehensive evaluation of cell morphology, transcriptomic expression patterns, and protein expression levels, with a focus on immune‐related components and the validation of immune function. Our study highlights the critical role of the source of the cell line in immunological research, demonstrating the development of different THP‐1 subtypes due to prolonged culture under various conditions. Previous studies by Kasai et al. [16], Kohro et al. [15], and Noronha et al. [17] have documented such alterations within the THP‐1 cell line, primarily focusing on genomic and transcriptomic variations. To our knowledge, we are the first to extend the comparison to the differentiation states of THP‐1 cells, specifically M1 and M2 macrophages, and to further expand the study by analyzing protein expression of immune‐relevant components, metabolic comparison, and assessing the immune functional potential. Notably, our results revealed that experimental outcomes can diverge significantly even when using the same cell line from different depositors with identical experimental setups. This variability underscores the importance of standardizing cell line characterization to ensure the reliability and reproducibility of experimental results. To contextualize these findings, we compared both THP‐1 subtypes to a primary human immune cell setup. While the two subtypes differed from each other, they both shared a high degree of transcriptomic and immunological similarity with the primary monocyte/macrophage system, especially after differentiation. These results suggest that, despite depositor‐related variability, both THP‐1 subtypes are suitable models for studying immune responses. It has to be acknowledged that THP‐1 cells are a very valuable and widely used cell model but represent, as any in vitro model, merely a simplified approximation of in vivo biology.

## Author Contributions

**Sabine Klatt**: methodology, investigation, formal analysis, writing – original draft. **Gudrun Marquardt**: data curation, visualization, formal analysis, writing – review and editing. **Miriam Faxel**: data curation, visualization, formal analysis, writing – review and editing. **Stefan Rubner**: supervision, project administration, conceptualization, methodology, writing – review and editing. **Ioannis Papasotiriou**: project administration, resources, writing – review and editing.

## Funding

This research did not receive any specific grant from funding agencies in the public, commercial, or nonprofit sector and was conducted as part of the authors’ occupation at Research Genetic Cancer Centre Central Europe GmbH (S.K., G.M., M.F., and S.R.). and Research Genetic Cancer Centre International GmbH (I.P.), respectively.

## Conflicts of Interest

The authors declare no conflicts of interest.

## Supporting Information

Additional supporting information can be found online in the Supporting Information section.

## Supporting information


**Supporting Information** Figure S1: Transcriptomic profiles of THP‐1 cells from both depositors determined by microarray. Comparable to Figure 2, the transcriptome was analyzed by microarray. (A) Volcano plots visualize upregulated (red, p.adj. ≤ 0.05, log2(FC) > 1) and downregulated (blue, p.adj. ≤ 0.05, log2(FC) ≤ –1) genes in each immune state comparing II to I. (B) VENN diagrams visualizing the overlap of upregulated and downregulated genes across different immune states between the two depositors. (C) Functional gene set enrichment analysis based on the Hallmark database revealing activated (red) and suppressed (blue) pathways for depositor II compared to I. (D) Heatmap presenting the log2(FC) of the inflammatory gene set for each differentiation state comparing depositor II to I with statistically significant difference displayed as star (*p* ≤ .05). Figure S2: VENN diagram displaying the overlap of expressed genes in THP‐1 cells I and II. Figure S3: Different protein abundance of immune‐related cell surface markers CD16, CD32 and CD45 during differentiation compared to mRNA levels. A‐C) mRNA expression levels of CD16, CD32 with both transcripts, and CD45 based on TPMs. D‐F) Representative flow cytometric histograms visualize the percentage of cells presenting the cell surface markers CD16, CD32 and CD45. The corresponding isotype controls are shown as dotted lines. Bar charts depict the mean ± SD (*n* = 4). Statistical significance was calculated using two‐way ANOVA with subsequent Tukey post hoc test for multiple comparison correction ( ^∗^ *p* ≤ .05,  ^∗∗^ *p* ≤ .01,  ^∗∗∗^ *p* ≤ .001,  ^∗∗∗∗^ *p* ≤ .0001). Figure S4: Different protein abundance of immune‐related cell surface markers CD163 and CD182 during differentiation compared to mRNA levels. (A–B) mRNA expression levels of CD163 and CD182 based on TPMs. (C–D) Representative flow cytometric histograms visualize the percentage of cells presenting the cell surface markers CD163 and CD182. The corresponding isotype controls are shown as dotted lines. Bar charts depict the mean ± SD (*n* = 4). Statistical significance was calculated using two‐way ANOVA with subsequent Tukey post hoc test for multiple comparison correction ( ^∗^ *p* ≤ .05, ^∗∗^ *p* ≤ .01,  ^∗∗∗^ *p* ≤ .001,  ^∗∗∗∗^ *p* ≤ .0001). Figure S5: Quantification profiles of secreted analytes in the supernatant during differentiation compared to mRNA levels. (A–C and G–I) mRNA expression level based on TPMs. (D–F and J–L) Concentrations of secreted analytes in supernatant samples. Bar charts depict mean ± SD (*n* = 4). Statistical significance was calculated using two‐way ANOVA with subsequent Tukey post hoc test for multiple comparison correction ( ^∗^ *p* ≤ .05,  ^∗∗^ *p* ≤ .01,  ^∗∗∗^ *p* ≤ .001,  ^∗∗∗∗^ *p* ≤ .0001). Figure S6: Transcriptomic profiles of THP‐1 cells from both depositors and primary monocytes with their differentiation states determined by microarray. Comparable to Figure 7, the transcriptome was analyzed by microarray. (A) Pearson correlogram of all samples indicating high correlation in dark blue and hierarchical clustering of the samples. (B) VENN diagrams showing overlap of expressed genes in primary immune cells (gray), depositor I (orange) and depositor II (green). (C) Gene set enrichment analysis based on hallmark gene sets showing activated (red) and suppressed (blue) pathways in the comparison of depositor I or II to primary immune cells. (D) Heatmap of inflammatory response pathway gene set in depositor I and II compared to the primary set‐up with statistically significant difference displayed as star (*p* ≤ .05). Figure S7: Clustering of THP‐1 cells of both depositors and primary monocytes with their differentiated states by PCA based on their transcriptome analyzed by RNAseq. Table S1: STR profile of THP‐1 cell line from depositor I and II showing genetic characteristic of both cell lines determined by PCR‐single locus‐technology using Thermo Fisher, AmpFISTR Identifier Plus PCR Amplification Kit performed by Eurofins Genomics. Table S2: Antibodies used for flow cytometry staining in this study. Supplementary method description: Gene expression analysis by microarray

## Data Availability

The transcriptomic data sets are available in the BioStudies database (https://www.ebi.ac.uk/biostudies), under accession numbers E‐MTAB‐17224 (microarray data) and E‐MTAB‐17225 (RNA sequencing data).
