# Supplementary material for: Comparison of THP‐1 Subtypes Reveals Differences on Transcriptomic and Protein Expression Level Throughout Their Immunological Differentiation
Source: J Immunol Res. 2026 Aug 17;2026:9427482. doi: 10.1155/jimr/9427482 (PMC13478801; doi:10.1155/jimr/9427482)
Supplement: Supplementary file 1 [file JIMR-2026-9427482-s001.pdf]

Supplementary information

Supplementary figures

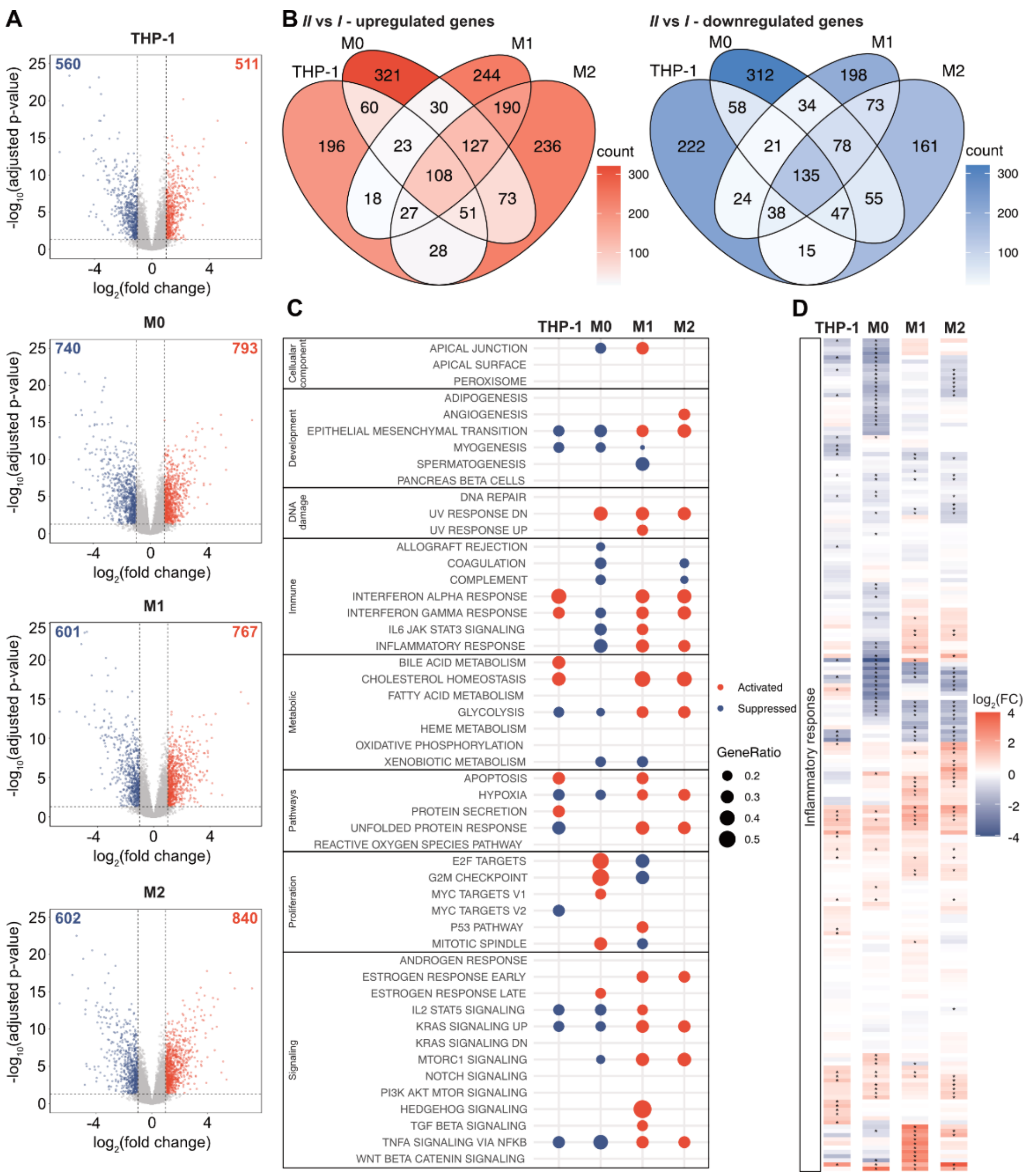

Figure S1: Transcriptomic profiles of THP-1 cells from both depositors determined by microarray. Comparable to Figure 2, the transcriptome was analyzed by microarray. A) Volcano plots visualize upregulated (red, p.adj. ≤ 0.05, $log_2(FC) > 1$) and downregulated (blue, p.adj. ≤ 0.05, $log_2(FC) \leq -1$) genes in each immune state comparing *II* to *I*. B) VENN diagrams visualizing the overlap of upregulated and downregulated genes across different immune states between the two depositors. C) Functional gene set enrichment analysis based on the Hallmark database revealing activated (red) and suppressed (blue) pathways for depositor *II* compared to *I*. D) Heatmap presenting the $log_2(FC)$ of the inflammatory gene set for each differentiation state comparing depositor *II* to *I* with statistically significant difference displayed as star (p.adj ≤ .05).

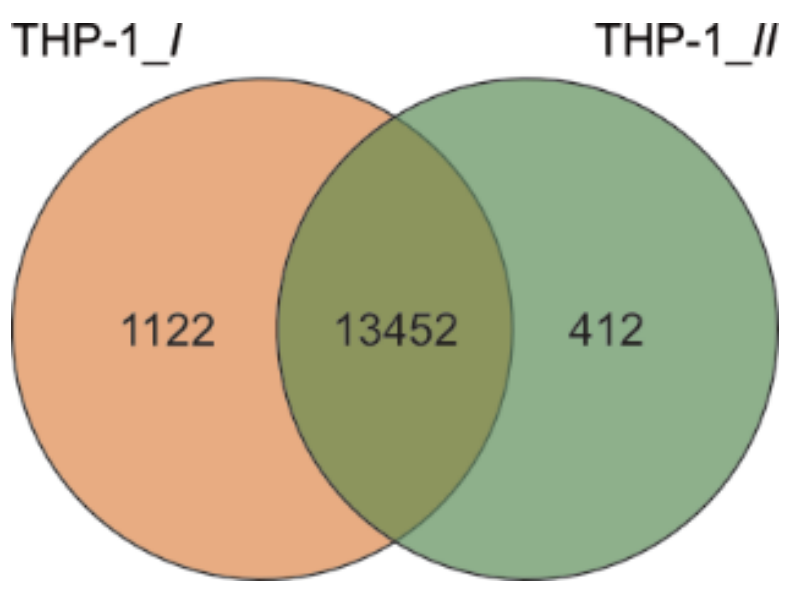

Figure S2: VENN diagram displaying the overlap of expressed genes in THP-1 cells *I* and *II*.

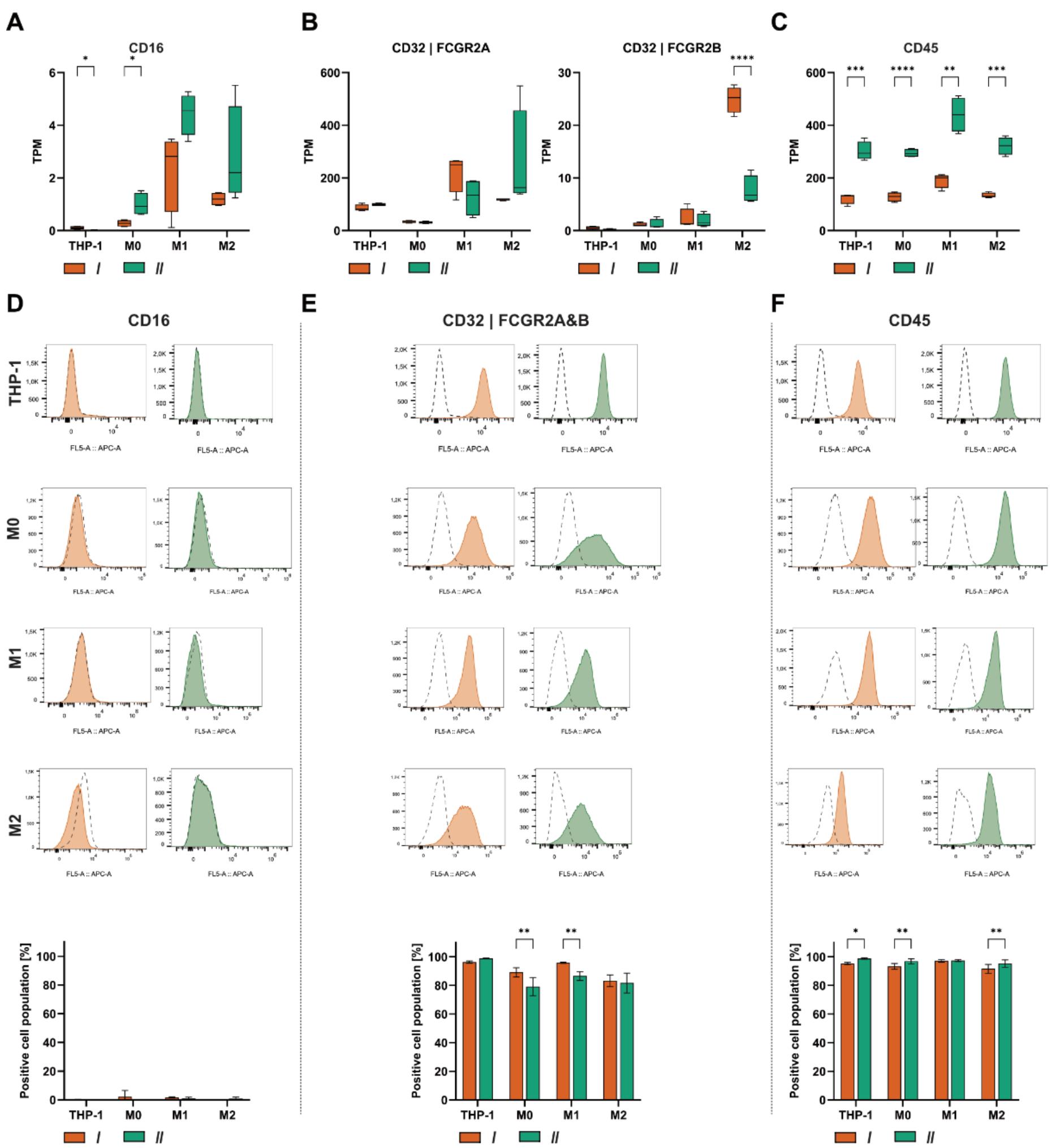

Figure S3: Different protein abundance of immune-related cell surface markers CD16, CD32 and CD45 during differentiation compared to mRNA levels. A-C) mRNA expression levels of CD16, CD32 with both transcripts, and CD45 based on TPMs. D-F) Representative flow cytometric histograms visualize the percentage of cells presenting the cell surface markers CD16, CD32 and CD45. The corresponding isotype controls are shown as dotted lines. Bar charts depict the mean ± SD (n=4). Statistical significance was calculated using two-way ANOVA with subsequent Tukey posthoc test for multiple comparison correction (* p ≤ .05; ** p ≤ .01; *** p ≤ .001, **** p ≤ .0001).

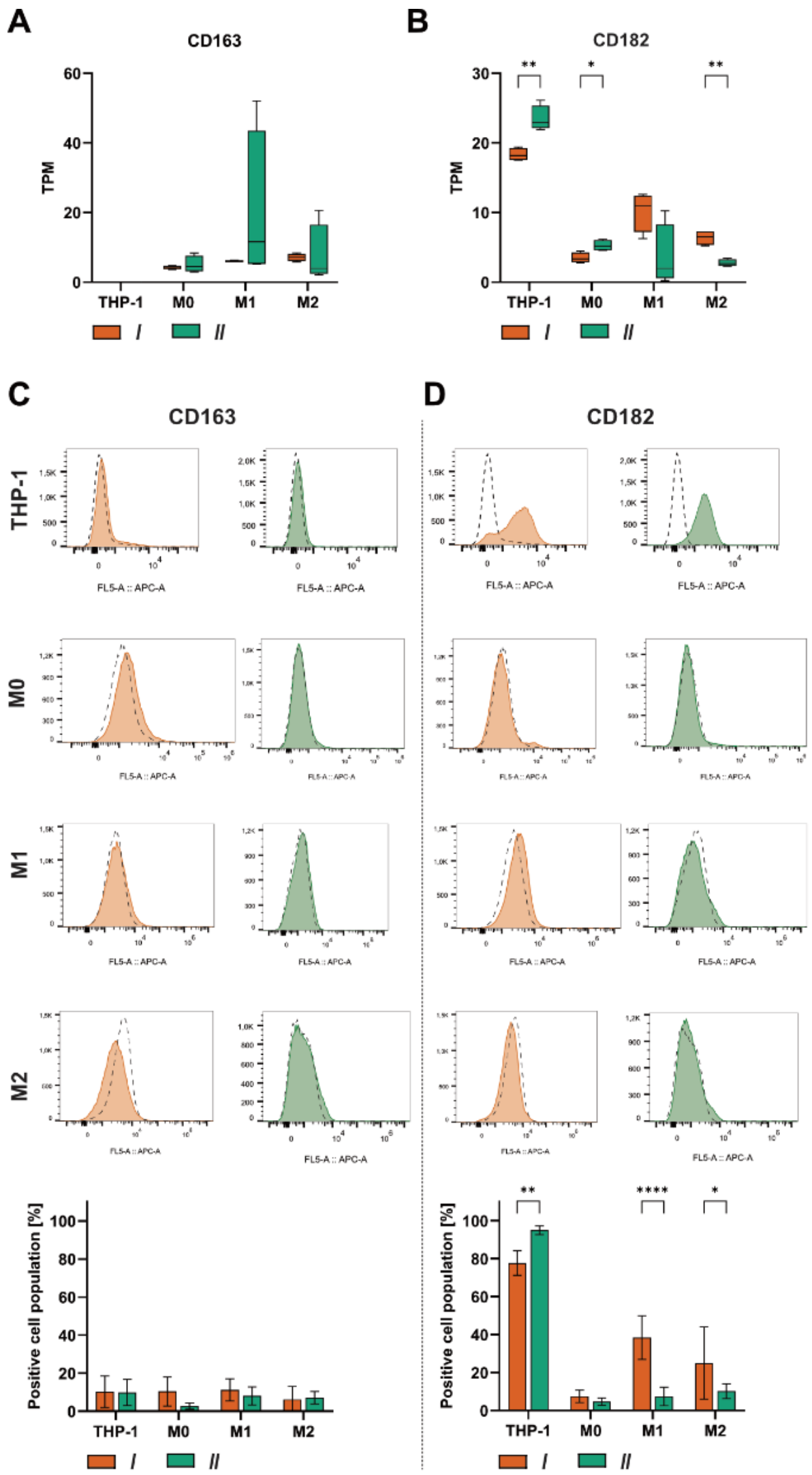

Figure S4: Different protein abundance of immune-related cell surface markers CD163 and CD182 during differentiation compared to mRNA levels. A-B) mRNA expression levels of CD163 and CD182 based on TPMs. C-D) Representative flow cytometric histograms visualize the percentage of cells presenting the cell surface markers CD163 and CD182. The corresponding isotype controls are shown as dotted lines. Bar charts depict the mean ± SD (n=4). Statistical significance was calculated using two-way ANOVA with subsequent Tukey posthoc test for multiple comparison correction (* p ≤ .05; ** p ≤ .01; *** p ≤ .001, **** p ≤ .0001).

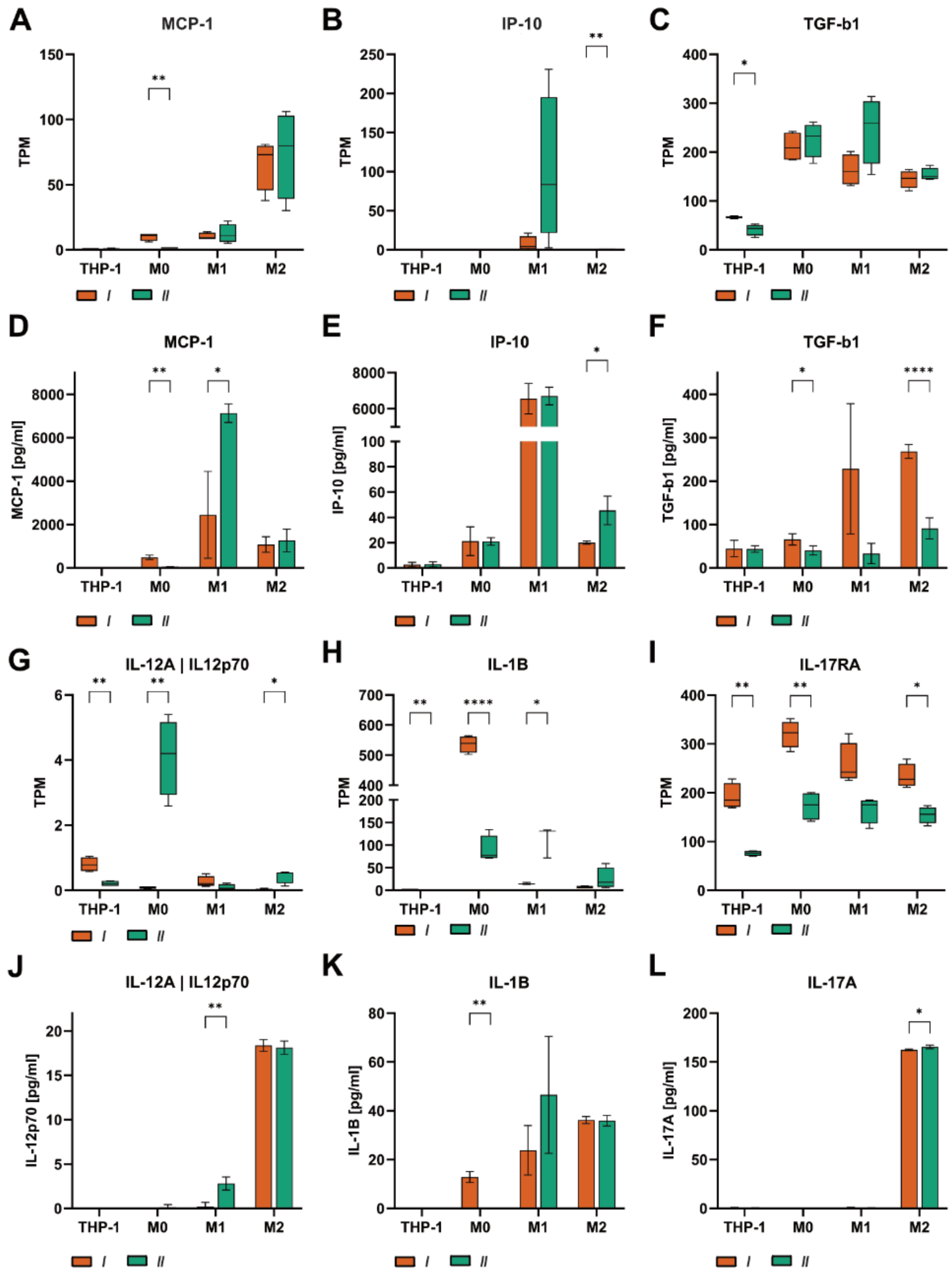

Figure S5: Quantification profiles of secreted analytes in the supernatant during differentiation compared to mRNA levels. A-C and G-I) mRNA expression levels based on TPMs. D-F and J-L) Concentrations of secreted analytes in supernatant samples. Bar charts depict mean ± SD (n=4). Statistical significance was calculated using two-way ANOVA with subsequent Tukey posthoc test for multiple comparison correction (* $p \leq .05$; ** $p \leq .01$; *** $p \leq .001$, **** $p \leq .0001$).

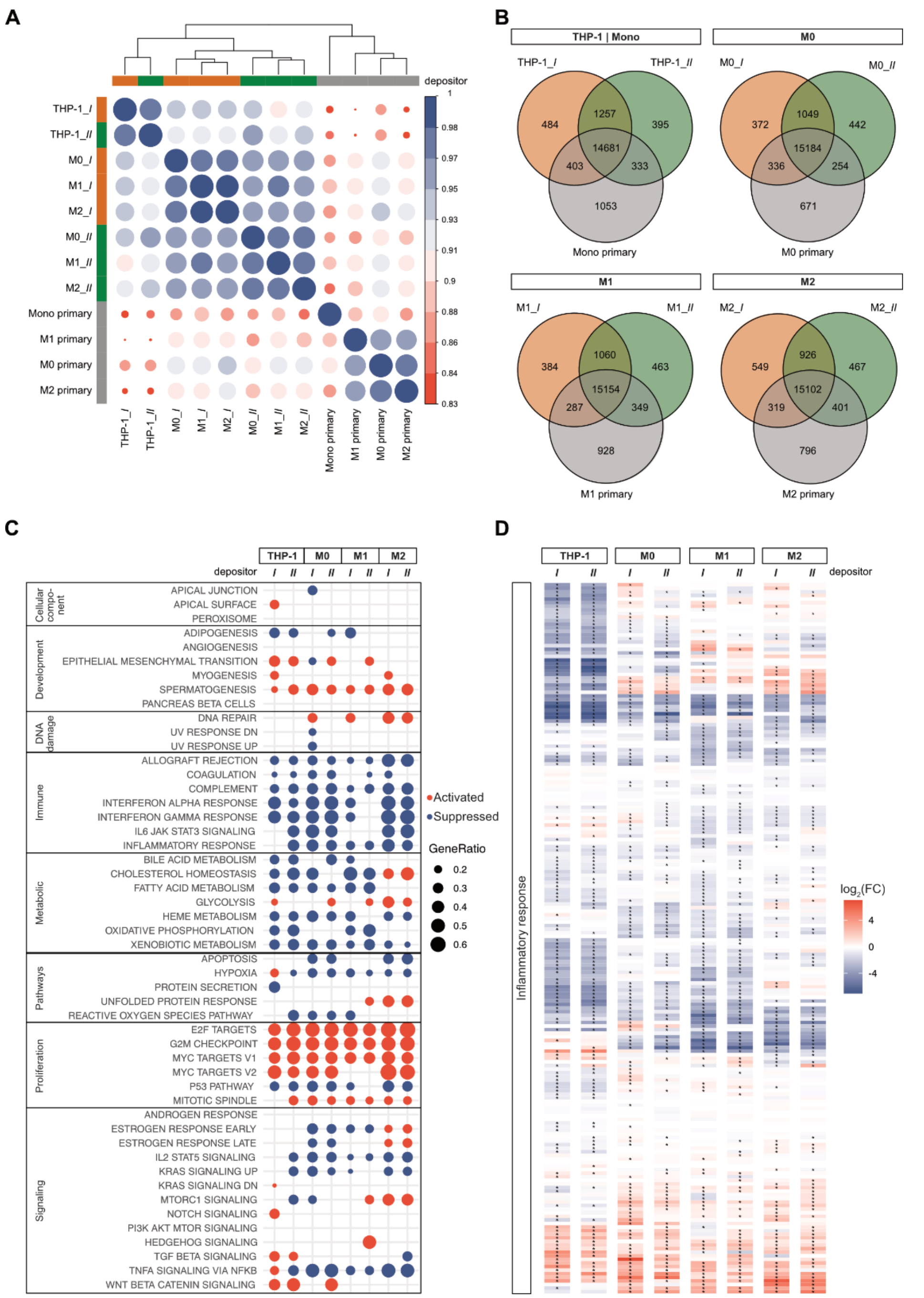

Figure S6: Transcriptomic profiles of THP-1 cells from both depositors and primary monocytes with their differentiation states determined by microarray. Comparable to Figure 7, the transcriptome was analyzed by microarray. A) Pearson correlogram of all samples indicating high correlation in dark blue and hierarchical clustering of the samples. B) VENN diagrams showing overlap of expressed genes in primary immune cells (grey), depositor *I* (orange) and depositor *II* (green). C) Gene set enrichment analysis based on hallmark gene sets showing activated (red) and suppressed (blue) pathways in the comparison of depositor *I* or *II* to primary immune cells. D) Heatmap of inflammatory response pathway gene set in depositor *I* and *II* compared to the primary set-up with statistically significant difference displayed as star (p.adj ≤ .05).

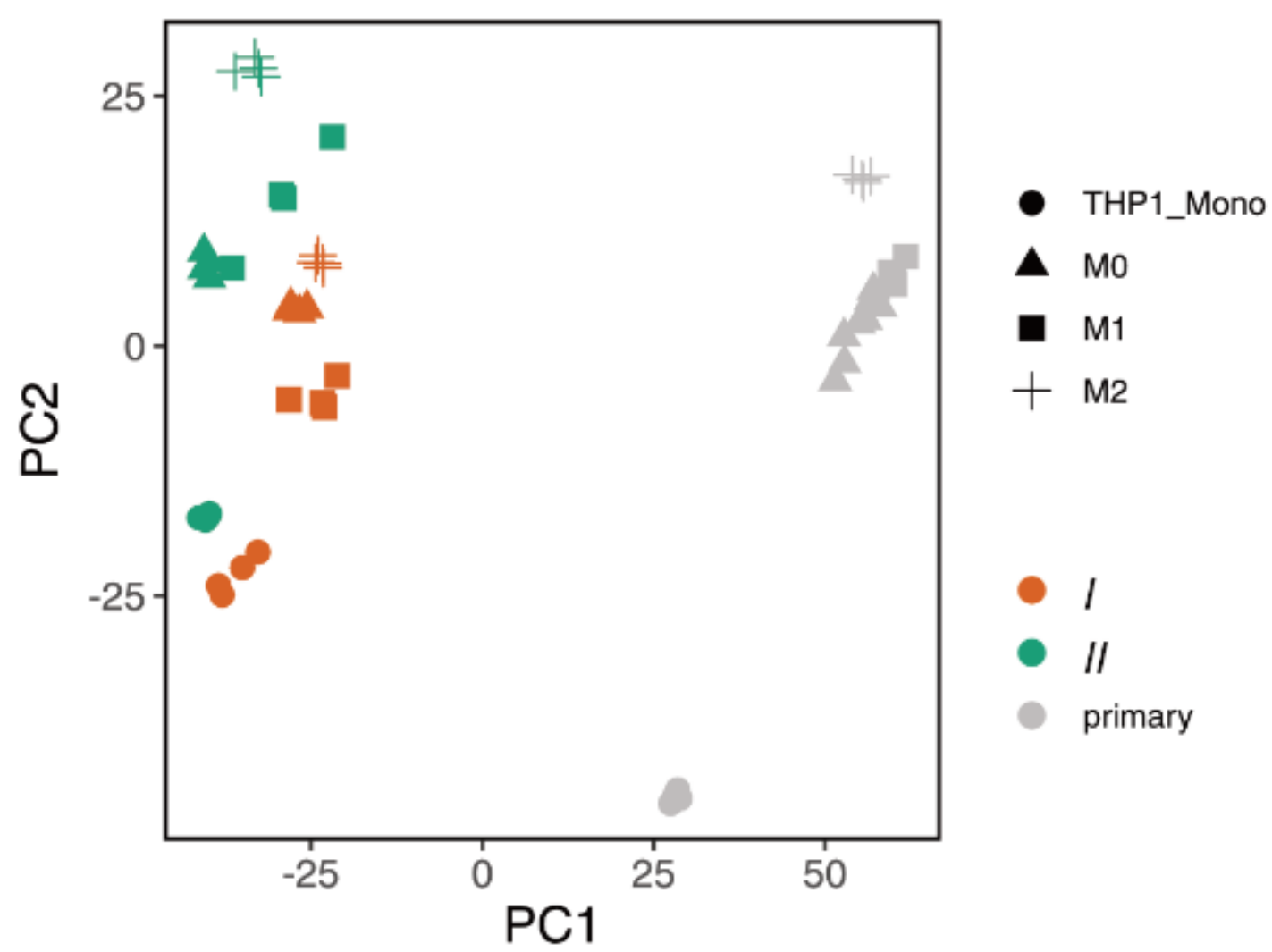

Figure S7: Clustering of THP-1 cells of both depositors and primary monocytes with their differentiated states by PCA based on their transcriptome analyzed by RNAseq.

Supplementary tables

Table S1: STR profile of THP-1 cell line from depositor *I* and *II* showing genetic characteristic of both cell lines determined by PCR-single locus-technology using Thermo Fisher, AmpFlSTR Identifier Plus PCR Amplification Kit performed by Eurofins Genomics.

	THP-1_*I*	THP-1_*II*
D8S1179	10,14	10,14
D21S11	30,31.2	30,31.2
D7S820	10,10*	10,10
CSF1PO	11,13	11,13
D3S1358	15,17	15,17
TH01	8,9.3	8,9.3
D13S317	8,13	13,13
D16S539	11,12	11,12
D2S1338	17,18	17,18
D19S433	12.2,13	12.2,13
vWA	16,17	16,16
TPOX	8,11	8,11
D18S51	13,14	13,14
AMEL	X,Y	X,Y
D5S818	11,12	11,12
FGA	24,25	24,25

Table S2: Antibodies used for flow cytometry staining in this study.

	Supplier	Catalog #	Clone
APC Mouse IgG1, κ Isotype Ctrl (FC) Antibody	Biolegend	400122	MOPC-21
APC Mouse IgG2b, κ Isotype Ctrl Antibody	Biolegend	400322	MPC-11
APC anti-human CD86 Antibody	Biolegend	374207	BU63
APC anti-human CD16 Antibody	Biolegend	360705	B73.1
APC anti-human CD182 (CXCR2) Antibody	Biolegend	320710	5E8/CXCR2
APC anti-human CD163 Antibody	Biolegend	326509	RM3/1
APC anti-human CD45 Antibody	Biolegend	304011	HI30
APC anti-human CD14 Antibody	Biolegend	325607	HCD14
APC anti-human CD206 (MMR) Antibody	Biolegend	321109	15-2
APC anti-human CD209 (DC-SIGN) Antibody	Biolegend	343005	DCS-8C1
APC anti-human CD32 Antibody	Biolegend	303207	FUN-2

Supplementary method description

Gene expression analysis by microarray

The transcriptome-wide profiles were conducted using a one-color microarray gene expression analysis as described by Agilent. Briefly, 200 ng of total RNA were reverse-transcribed to cDNA, further transcribed to cRNA and labeled with Cyanin 3-CTP via the Low Input Quick Amp Labeling Kit (Agilent, 5190-2305). Labeled cRNA was purified by using the RNeasy Mini kit (Qiagen) without on-column DNase digest. 600 ng purified cRNA was fragmented according to the Gene Expression Hybridization Kit (Agilent, 5188-5242) and transferred to Gasket slide (Agilent, G2534-60014). The microarray chip SurePrint G3 human gene expression 8x60k v3 (Agilent, G4851C) was added to Gasket slide to form the hybridization sandwich which was fixed via the hybridization chamber (Agilent, G2534A) and subsequently incubated at 65 °C for 17 h while rotation. Unbound cRNA was removed using the Gene Expression Wash Buffer Kit (Agilent, 5188-5327) followed by immediate scanning of the prepared microarray chip via microarray scanner InnoScan910 (Innopsys). InnoScan910 controlling and raw data extraction was performed via the Mapix software (Innopsys, commercial license).

Microarray data were analyzed using R [1] and Bioconductor [2, 3] with the following associated packages: biomaRt [4, 5], dendextend [6], dplyr [7], fgsea [8], ggsci [9], limma [10], msigdb [11], tidyverse [12], rtracklayer [2] and visualized using ggplot2 [13], ggVennDiagram [14], ggthemes [15]. Raw intensities were log2-transformed, background corrected using the normexp method and an offset of 50, and quantile normalized. Microarray probes were filtered for protein-coding genes and for probes expressed (> above the 90 % of the negative control) in at least one sample group. Identical probes and those representing the same gene, were aggregated by averaging their intensity values using the limma::avereps function. The gene annotation release 110 by ensembl [16] was used throughout the analysis. Differentially expressed genes were identified by fitting linear models (limma package) and using moderated t-statistics and Benjamini and Hochberg's p-value adjustment [17].

Subsequently, enriched biological pathways were determined by using gene set enrichment analysis [18], [19] based on the hallmark gene sets by the Molecular Signature Database (MSigDb) [20].

References

[1] R. Team, "R: A language and environment for statistical computing.," *MSOR Connect.*, 2014, Accessed: Sep. 05, 2023. [Online]. Available: https://www.semanticscholar.org/paper/R%3A-A-language-and-environment-for-statistical-Team/659408b243cec55de8d0a3bc51b81173007aa89b

[2] R. C. Gentleman *et al.*, "Bioconductor: open software development for computational biology and bioinformatics," *Genome Biol.*, vol. 5, no. 10, p. R80, Sep. 2004, doi: 10.1186/gb-2004-5-10-r80.

[3] W. Huber *et al.*, "Orchestrating high-throughput genomic analysis with Bioconductor," *Nat. Methods*, vol. 12, no. 2, Art. no. 2, Feb. 2015, doi: 10.1038/nmeth.3252.

[4] S. Durinck, P. T. Spellman, E. Birney, and W. Huber, "Mapping identifiers for the integration of genomic datasets with the R/Bioconductor package biomaRt," *Nat. Protoc.*, vol. 4, no. 8, Art. no. 8, Aug. 2009, doi: 10.1038/nprot.2009.97.

[5] S. Durinck *et al.*, "BioMart and Bioconductor: a powerful link between biological databases and microarray data analysis," *Bioinformatics*, vol. 21, no. 16, pp. 3439–3440, Aug. 2005, doi: 10.1093/bioinformatics/bti525.

[6] T. Galili, "dendextend: an R package for visualizing, adjusting and comparing trees of hierarchical clustering," *Bioinformatics*, vol. 31, no. 22, pp. 3718–3720, Nov. 2015, doi: 10.1093/bioinformatics/btv428.

[7] H. Wickham, R. François, L. Henry, K. Müller, and D. Vaughan, *dplyr: A Grammar of Data Manipulation*. (2023). [Online]. Available: https://CRAN.R-project.org/package=dplyr

[8] G. Korotkevich, V. Sukhov, N. Budin, B. Shpak, M. Artyomov, and A. Sergushichev, "Fast gene set enrichment analysis," 2016. doi: 10.1101/060012.

[9] N. Xiao, *ggsci: Scientific Journal and Sci-Fi Themed Color Palettes for "ggplot2."* (2023). [Online]. Available: https://nanx.me/ggsci/

[10] M. E. Ritchie *et al.*, "limma powers differential expression analyses for RNA-sequencing and microarray studies," *Nucleic Acids Res.*, vol. 43, no. 7, p. e47, Apr. 2015, doi: 10.1093/nar/gkv007.

[11] D. Bhuva, G. Smyth, and A. Garnham, *msigdb: An ExperimentHub Package for the Molecular Signatures Database (MSigDB)*. (2022). [Online]. Available: https://davislaboratory.github.io/msigdb

[12] H. Wickham *et al.*, "Welcome to the Tidyverse," *J. Open Source Softw.*, vol. 4, no. 43, p. 1686, Nov. 2019, doi: 10.21105/joss.01686.

[13] H. Wickham, *ggplot2*. in Use R! Cham: Springer International Publishing, 2016. doi: 10.1007/978-3-319-24277-4.

[14] C. Gao, *ggVennDiagram: A "ggplot2" Implement of Venn Diagram*. (2022). [Online]. Available: https://CRAN.R-project.org/package=ggVennDiagram

[15] J. Arnold, *ggthemes: Extra Themes, Scales and Geoms for "ggplot2."* (2021). [Online]. Available: https://CRAN.R-project.org/package=ggthemes

[16] S. C. Dyer *et al.*, "Ensembl 2025," *Nucleic Acids Res.*, vol. 53, no. D1, pp. D948–D957, Dec. 2024, doi: 10.1093/nar/gkae1071.

[17] Y. Benjamini and Y. Hochberg, "Controlling the False Discovery Rate: A Practical and Powerful Approach to Multiple Testing," *J. R. Stat. Soc. Ser. B Methodol.*, vol. 57, no. 1, pp. 289–300, 1995, doi: 10.1111/j.2517-6161.1995.tb02031.x.

[18] V. K. Mootha *et al.*, "PGC-1α-responsive genes involved in oxidative phosphorylation are coordinately downregulated in human diabetes," *Nat. Genet.*, vol. 34, no. 3, Art. no. 3, Jul. 2003, doi: 10.1038/ng1180.

[19] A. Subramanian *et al.*, "Gene set enrichment analysis: A knowledge-based approach for interpreting genome-wide expression profiles," *Proc. Natl. Acad. Sci.*, vol. 102, no. 43, pp. 15545–15550, Oct. 2005, doi: 10.1073/pnas.0506580102.

[20] A. Liberzon, C. Birger, H. Thorvaldsdóttir, M. Ghandi, J. P. Mesirov, and P. Tamayo, "The Molecular Signatures Database (MSigDB) hallmark gene set collection," *Cell Syst.*, vol. 1, no. 6, pp. 417–425, Dec. 2015, doi: 10.1016/j.cels.2015.12.004.
